# A First-Order Shear Deformation Theory-Based Analytical Approach for Acoustic-Vibration Research of Rib-Stiffened PVC Foam Sandwich Structures with Reinforcing and Weakening Phases

**DOI:** 10.3390/polym18080910

**Published:** 2026-04-08

**Authors:** Zhaozhe Ma, Ruijie Dai, Zhiwei Zhou, Ying Li

**Affiliations:** 1 School of Naval Architecture, Ocean and Energy Power Engineering, Wuhan University of Technology, Wuhan 430070, China; 13395594994@163.com; 2 Wuhan Second Ship Design and Research Institute, Wuhan 430205, China; hust_dairj@163.com; 3 Beijing Key Laboratory of Lightweight Multi-Functional Composite Materials and Structures, Institute of Advanced Structure Technology, Beijing Institute of Technology, Beijing 100080, China

**Keywords:** rib-stiffened PVC foam sandwich structures, reinforcing and weakening phases, underwater environment, analytical approach, acoustic vibration analyses

## Abstract

This paper presents a theoretical approach based on the FSDT to study the acoustic vibration performance of rib-stiffened PVC foam sandwich structures with reinforcing and weakening phases when submerged in water. The complex core layer with reinforcing and weakening phases is homogenized to an equivalent orthotropic layer. Building upon this framework, the governing equations of motion for rib-stiffened PVC foam sandwich structures under the boundary conditions of a simply supported type are derived, incorporating the coupling interaction between the reinforcing ribs and the sandwich plates. Considering the influence of the underwater environment, with the Helmholtz equation governing the continuity of the acoustic pressure field and the Euler equation regulating the fluid–structure interaction interface continuity, the Navier method is subsequently employed to solve for the natural frequencies and acoustic vibration responses. For the purpose of verifying the proposed approach, the predicted results are contrasted with both the literature-derived data and numerical simulation results. Finally, parametric research is further conducted to explore the effect of the parameters of the rib and core layers on the underwater acoustic vibration characteristics. The conclusions drawn from this study can provide meaningful guidance for engineering design and optimization of such rib-stiffened sandwich structures, incorporating both reinforcing and weakening phases in underwater engineering applications.

## 1. Introduction

The sandwich structures integrated with both generalized reinforcing and weakening phases have found widespread applications in aerospace, automotive, marine, and other related engineering fields owing to their excellent performance characteristics, including their remarkable strength-to-weight characteristics, excellent anti-corrosion performance, and acoustic function design flexibility [1,2]. The reinforcing phases include metal particles and glass/carbon fiber-enhanced lattice columns, trusses, and honeycomb partitions [3,4,5]. The weakened phases generally contain porous sound-absorbing or sound-insulating foam, a soft vibration damping layer, an acoustic functional cavity, and so on [6,7]. In marine engineering, the rib-stiffened PVC foam sandwich structures with reinforcing and weakening phases could be applied on superstructures, such as the sail and rudder plate of an underwater vehicle. With an appropriate acoustic function design, the sandwich structure could gain excellent performances of vibration attenuation and noise isolation or absorption [8,9], as well as its lightweight and high rigidity characteristics. Once excited by the external load in the underwater environment, it is imperative to investigate the acoustic vibration characteristics of the rib-stiffened sandwich in acoustic-structural coupling analysis.

The research on composite structures has a long history, among which the most representative theories are the CLT (classical lamination theory) and FSDT (first-order shear deformation theory) [10]. The CLT assumes that plane sections remain plane and normal to the mid-surface after deformation, which is well-suited for thin composite plates where shear deformation effects are negligible [11,12,13]. The FSDT, by contrast, incorporates the role of rotary inertia and shear deformation, rendering it more appropriate for moderately thick composite plates [14,15,16]. These classical theories have not only elucidated fundamental mechanical responses of composite plates but have also been widely adopted as essential tools in the preliminary design and performance analysis of composite plate structures across various engineering domains. Based on the CLT and the FSDT, scholars have developed and derived various higher-order shear deformation theories (HSDTs). Tu et al. [17] adopted the third-order shear deformation theory (TSDT) as the theoretical basis and derived the calculation formula for the driving force of macroscopic fiber composite materials. To conduct static mechanical analysis of functionally graded saturated porous plates resting on the Paschen–Narkovsky elastic foundation, Tru et al. [18] proposed a novel quasi-3D-HSDT and employed it for the analysis. Lakhemissi et al. [19] used the integral-type-HSDT, in consideration of the transverse shear effect, to investigate the mechanical behavior of bidirectional functionally graded materials plates under elastic bending conditions. As an expansion of the CLT and the FSDT, HSDTs are widely applied in the field of composite laminated plate design. Compared with traditional theories, it could make more accurate predictions of structural stress and deformation.

Over the past few decades, researchers have conducted extensive research on the mechanical and acoustic properties of rib-stiffened homogeneous plates. Hou et al. [20] conducted experiments and numerical methods to study the low-speed impact (LVI) and post-impact compression (CAI) behaviors of rib-strengthened CFRP panels. In the stability analysis of U-shaped rib-stiffened plates, Wang et al. [21] explored how aspect ratios affect stability performance via tests and numerical simulations, and put forward reasonable width-to-height ratio recommendations for engineering practice. Yekta et al. [22] derived an analytical method by solving the generalized Bragg wave incidence, which was used to consider the acoustic properties of multiple rib-stiffened plates connected in series. Chen et al. [23] investigated the influence of varying welding energy inputs on the vibro-acoustic characteristics of stiffened plate structures, performing a series of experiments on the vibration modes, underwater vibration responses, and acoustic radiation behaviors of such structures under different welding energy input conditions. In conclusion, the current extensive research on rib-stiffened plates mainly focuses on homogeneous structures. By combining experimental methods, numerical simulations, and analytical methods, the influences of the structural parameters, loading conditions, and processing techniques on the mechanical properties and acoustic characteristics of the structure have been systematically investigated, providing data and theoretical support for the design optimization of the base reinforcement plate structure.

To meet the comprehensive requirements of underwater equipment, such as lightweighting, high strength, shock absorption, and pressure resistance, researchers have gradually broken through the limitations of homogeneous structures and achieved improvements in structural performance by introducing reinforcing phases in the core. Roun et al. [24] proposed a new type of nanocomposite material, which was used to integrate GPLs into the plate structure of a traditional functional graded matrix, and the performance of such plates was evaluated through free vibration and buckling analyses. Wang et al. [25] combined topological optimization to design three bimodal lattice/CFRP sandwich structures. Through testing the bending behavior and damage process, they revealed the complex failure modes and energy absorption enhancement mechanisms of this type of structure. Leveraging the interfacial stress transfer mechanism between the fiber-reinforced composite layer, damping layer, and reinforcing ribs, Wang et al. [26] proposed an innovative dynamic analytical model to investigate the dynamic performance of rib-reinforced composite damping plates integrated with randomly aligned carbon nanotubes. Madenci et al. [27,28] investigated the bending behavior of FG-CNTRC sandwich beams with a pultruded GFRP core, revealing the influence of the pultruded composite core on the bending mechanical response of the sandwich beam; meanwhile, systematic experimental and analytical studies on the buckling performance of FG-CNTRC sandwich beams have also been carried out, and the critical buckling load and failure mode of such structures have been clarified with a verifiable analytical prediction model. Most of the current research on enhanced phase-related studies focus on the modification of a single reinforcing phase and mostly target the optimization of the mechanical properties of the structures in an air environment. The research on the acoustic vibration coupling characteristics of rib-stiffened composite structures containing reinforcing phases in underwater environments is relatively insufficient, and there is even less involvement in the coordinated regulation with other functional phases.

Unlike the reinforcing phases, which aim to improve the structural mechanical load-bearing capacity and vibration resistance stiffness as its main goal, the introduction of the functional weakening phases are guided by the core objective of actively dissipating vibration energy and optimizing the acoustic radiation characteristics [29]. Kong et al. [30] investigated a butterfly-like double-layer Helmholtz resonator structure lined with porous materials. By virtue of a distinctive resonant mechanism, this structure enabled efficient acoustic wave absorption at specific frequencies, thus achieving effective ambient noise attenuation. Wang et al. [31] designed a sound-absorbing periodically arrayed structure (SPAS) by leveraging the synergistic effects of cavity resonance and impedance transition. This structure exhibited a sound absorption coefficient of 0.9 within the frequency range of 2400–10,000 Hz at a water pressure of 1.5 MPa, and further realized broadband sound absorption at a water depth of 300 m. Liu et al. [32] proposed a new type of Helmholtz resonant metamaterial composed of perforated rigid panels and perforated porous materials. This structure dissipated acoustic energy via the porous material lining the back cavity, enabling nearly perfect sound absorption across a broad range of neck diameters. In the field of bio-inspired sandwich structures, Song et al. [33,34] studied the acoustic properties of honeycomb sandwich panels filled with paper fibers/cement panels, and conducted an in-depth analysis of the effects of the filling material, panel configuration, and core material type on the FHW sound insulation performance. In addition, the vibration characteristics of the foam-filled Beetle elytron plate (BEP) were also investigated by changing the structural parameters.

Most current studies have mainly focused on rib-stiffened homogeneous plates and sandwich plates that contain only a single reinforcing phase or a single weakening phase; however, there is still a lack of research on the underwater acoustic vibration response characteristics of composite plates that have both rib stiffeners and simultaneously contain reinforcing and weakening phases. Such composite plates have broad application potential in the field of ship engineering. However, due to their complex form and the diverse components of the core layer materials, vibration analyses of these composite plates is difficult to conduct, except in cumbersome specific structural refinement modeling based on numerical simulations.

To deal with the problem, this paper has constructed a theoretical method applicable to analyzing the acoustic vibration characteristics of rib-stiffened PVC foam sandwich structures with reinforcing and weakening phases in underwater environments. The composite core containing reinforcing and weakening phases is equivalently represented as a single orthotropic layer to simplify characterization. For the rib-stiffened sandwich under simply supported boundary constraints, the motion equations are derived by considering the coupling effect between the ribs and the sandwich plates. Regarding the coupling relationship between the underwater environment and the structure, the distribution of sound pressure fields is described by the Helmholtz equation, while the continuity condition on the fluid–solid interaction interface is controlled by the Euler equation. The control equations are solved using the Navier method to obtain the natural frequencies and acoustic vibration response. Parametric studies are further conducted to explore the influence of the parameters of the rib and core layers on the underwater acoustic vibration characteristics.

The main innovations of this paper are reflected in the following three aspects. First, by focusing on rib-stiffened PVC foam sandwich structures with both reinforcing and weakening phases, a novel theoretical method has been established to realize the rapid and accurate calculation of acoustic vibration response. Unlike the existing analytical methods, the proposed method achieves a balance between calculation efficiency and precision, effectively filling the gap in efficient analytical methods for such complex sandwich structures with dual-phase core defects. Furthermore, the proposed equivalent single-layer theoretical method possesses excellent universality. It can be flexibly extended to the acoustic vibration analysis of other composite structures with complex core layers, providing a unified theoretical framework for similar complex structure analyses. Finally, this paper conducts systematic variable analyses of material and geometric parameters for the rib-stiffened PVC foam sandwich structures with reinforcing and weakening phases. This not only reveals the intrinsic influence laws of key parameters on the acoustic vibration characteristics of such structures but also provides targeted theoretical references and reliable data support for future related studies and engineering design optimization, which has important engineering application value.

## 2. Analytical Method

### 2.1. Structural Characterization of the Rib-Stiffened Sandwich

**Figure 1:**
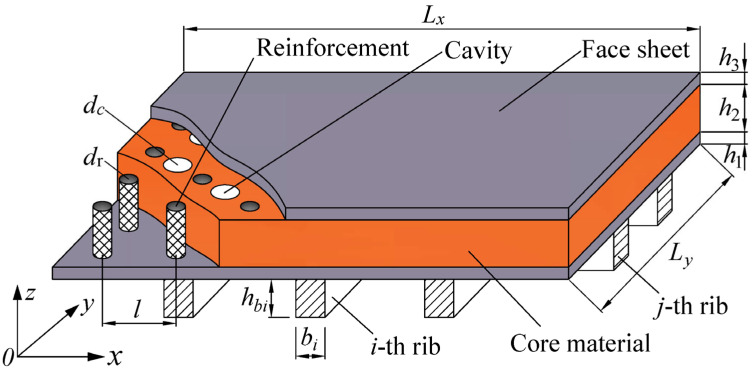
Geometry and dimension of orthogonal rib-stiffened PVC foam sandwich structures with reinforcing and weakening phases.

The geometry and corresponding dimensions of orthogonal rib-stiffened PVC foam sandwich structures with reinforcing and weakening phases are illustrated in Figure 1. In this composite structure, the reinforcement cylinders serve as the reinforcing phase, while the cylindrical cavities act as the functional weakening phases. The planar dimensions of the structure are specified as a length of $L_{x}$ and a width of $L_{y}$; meanwhile, the thicknesses corresponding to the lower face sheet, the core layer, and the upper face sheet are denoted by $h_{1}$, $h_{2}$, and $h_{3}$. The total thickness is $H=h_{1}+h_{2}+h_{3}$. The diameters of the reinforcements are expressed as $d_{r}$, and the diameters of cavities are indicated as $d_{c}$, with a uniform spacing of l between individual reinforcements or cavities. There are $n_{1}$ and $n_{2}$ rib stiffeners parallel to the *x*- and *y*-axes, respectively. The *i*-th rib is parallel to the *y*-axis and the *j*-th rib is parallel to the *x*-axis, as shown in the Figure 1 with $i=1,2,\ldots ,n_{2}$ and $j=1,2,\ldots ,n_{1}$. For rectangular section cases, the height and width of the *i*-th rib are $h_{bi}$ and $b_{i}$, and those of the *j*-th rib are $h_{bj}$ and $b_{j}$.

### 2.2. Homogenization of the Core Layer

Homogenization treatment is indispensable for the PVC foam core layer with reinforcing and weakening phases when conducting vibration characteristic analysis of the rib-stiffened sandwich. This treatment is then applied to derive its equivalent elastic constants. For the classical average-field approximation theory of two-phase composite materials, this study adopts the Mori–Tanaka method [35] to complete the above homogenization analysis. This method has a simple mathematical expression and explicit analytical solution:

(1) $$L_{h}^{-1}=\left[I+C_{i}{\left[L_{m}+(L_{i}-L_{m})(C_{i}I+C_{m}S)\right]}^{-1}(L_{m}-L_{i})\right]/L_{m}$$

For generally orthotropic materials, the stiffness tensor L takes the following form:

(2) $$L={\left[\begin{array}{cccccc} 1/E_{xx} & -\mu_{xy}/E_{yy} & -\mu_{xz}/E_{zz} & 0 & 0 & 0\\ -\mu_{yx}/E_{xx} & 1/E_{yy} & -\mu_{yz}/E_{zz} & 0 & 0 & 0\\ -\mu_{zx}/E_{xx} & -\mu_{zy}/E_{yy} & 1/E_{zz} & 0 & 0 & 0\\ 0 & 0 & 0 & 1/G_{yz} & 0 & 0\\ 0 & 0 & 0 & 0 & 1/G_{xz} & 0\\ 0 & 0 & 0 & 0 & 0 & \;1/G_{xy} \end{array}\right]}^{-1}$$

where $E_{xx}$, $E_{yy}$, and $E_{zz}$ denote the Young’s modulus along the three principal directions; $G_{xy}$, $G_{yz}$, and $G_{xz}$ represent the shear modulus; $\mu_{xy}$, $\mu_{xz}$, and $\mu_{yz}$ denote the primary Poisson’s ratios; $\mu_{yx}$, $\mu_{zx}$, and $\mu_{zy}$ correspond to the secondary Poisson’s ratios; C refers to the volume fraction of the inclusion phase, where the subscripts ‘i’, ‘m’, and ‘h’ indicate the inclusion phase, matrix phase, and homogenized material, respectively; I is the identity tensor; and S is the Eshelby tensor, which is determined by the geometric shape of the inclusion and the Poisson’s ratio $\mu_{m}$ of the matrix phase. For cylindrical inclusions, this tensor satisfies the following expression:

(3) $$S=\left[\begin{array}{cccccc} 0 & 0 & 0 & 0 & 0 & 0\\ \frac{\mu_{m}}{2\left(1-\mu_{m}\right)} & \frac{5-4\mu_{m}}{8\left(1-\mu_{m}\right)} & \frac{4\mu_{m}-1}{8\left(1-\mu_{m}\right)} & 0 & 0 & 0\\ \frac{\mu_{m}}{2\left(1-\mu_{m}\right)} & \frac{4\mu_{m}-1}{8\left(1-\mu_{m}\right)} & \frac{5-4\mu_{m}}{8\left(1-\mu_{m}\right)} & 0 & 0 & 0\\ 0 & 0 & 0 & \frac{3-4\mu_{m}}{4\left(1-\mu_{m}\right)} & 0 & 0\\ 0 & 0 & 0 & 0 & 1/2 & 0\\ 0 & 0 & 0 & 0 & 0 & 1/2 \end{array}\right]$$

However, the classical Mori–Tanaka method is only applicable to composite material systems containing a single inclusion phase and lacks the capability to directly determine the equivalent elastic constants of rib-stiffened PVC foam sandwich structures with reinforcing and weakening phases. To address this limitation, this paper adopts the two-level progressive homogenization strategy proposed in Ref. [36]. Firstly, with isotropic PVC foam core material as the matrix phase, the voids are equivalently regarded as weakly elastic isotropic inclusions with Poisson’s ratio consistent with that of the core material. The volume fraction of cavities is defined as the ratio of the total volume of cavities to the total volume of the core matrix and cavities. Through the Mori–Tanaka method, they are homogenized into an anisotropic material A. Then, using material A as the new matrix phase and the reinforcements as the inclusion phase, the volume fraction of reinforcements is defined as the ratio of the total volume of reinforcements to the total volume of material A and reinforcements. Following the Eshelby tensor assumption in the first-level homogenization, the Mori–Tanaka method is again applied to obtain an anisotropic material B, achieving the equivalent homogenization of the three-phase reinforced foam core with cavities and reinforcements. Through this process, the orthogonal rib-stiffened PVC foam sandwich structures with reinforcing and weakening phases is simplified to a standard orthogonal rib-stiffened sandwich structure.

### 2.3. Motion Equations of Rib-Stiffened Sandwich

#### 2.3.1. Constitutive Relations and Displacement Fields for Sandwich Structures

**Figure 2:**
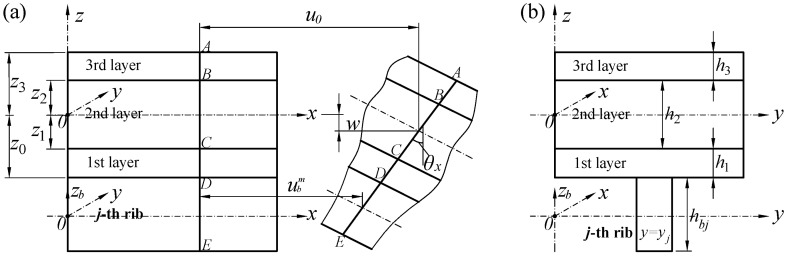
Deformations and coordinate system of the rib-stiffened sandwich: (**a**) *x*-*z* section; (**b**) *y*-*z* section.

The orthogonal rib-stiffened sandwich structure is made of a three-layer sandwich plate and ribs, as shown in Figure 2. To define the geometry of the sandwich plate, a global Cartesian coordinate system (*x*, *y*, *z*) is introduced, where the *x*-*y*-*zero* plane is positioned at the mid-plane of the structure. For each layer *k* (*k* = 1, 2, 3), the *z*-coordinates corresponding to its top and bottom interfaces are given by $z_{k}$ and $z_{k-1}$. The ribs are exhibited in local Cartesian coordinate systems. Taking the *j*-th rib parallel to *x*-axis as an example, there are the coordinates (*x*, *y*, *z_b_*) and *y* = *y_j_*, and the *x*-*y*-*zero* plane is located on the neutral plane of rib.

##### The Sandwich Plate

The mechanical behavior of the interlayer plate is described using FSDT, and the following simplified assumptions are introduced: (1) after deformation, the line segments that were perpendicular to the middle plane prior to deformation remain straight, though they are no longer perpendicular to the middle plane; and (2) prior to deformation, the line segments are perpendicular to the middle plane, they do not undergo axial elongation after deformation, and their points maintain consistent lateral displacements. Based on these assumptions, the displacement field (*U*, *V*, *W*) of the interlayer plate can be expressed as:

(4) $$\left\{\begin{array}{l} U\left(x,y,z,t\right)=u_{0}(x,y,t)+z\cdot \theta_{x}\left(x,y,t\right)\\ V\left(x,y,z,t\right)=v_{0}(x,y,t)+z\cdot \theta_{y}\left(x,y,t\right)\\ W\left(x,y,z,t\right)=w_{0}\left(x,y,t\right) \end{array}\right.$$

Based on the small deformation principle, the normal and shear strains are:

(5) $$\left\{\begin{array}{c} \varepsilon_{xx}\\ \varepsilon_{yy}\\ \gamma_{xy} \end{array}\right\}=\left\{\begin{array}{c} \varepsilon_{xx}^{m}+z\cdot \varepsilon_{xx}^{f}\\ \varepsilon_{yy}^{m}+z\cdot \varepsilon_{yy}^{f}\\ \gamma_{xy}^{m}+z\cdot \gamma_{xy}^{f} \end{array}\right\}\;,\;\;\left\{\begin{array}{c} \gamma_{yz}\\ \gamma_{xz} \end{array}\right\}=\left\{\begin{array}{c} \theta_{y}+w_{0,y}\\ \theta_{x}+w_{0,x} \end{array}\right\}$$

with

(6) $$\left\{\begin{array}{l} \varepsilon_{xx}^{m}=u_{0,x},\;\;\varepsilon_{xx}^{f}=\theta_{x,x}\\ \varepsilon_{yy}^{m}=v_{0,y},\;\;\varepsilon_{yy}^{f}=\theta_{y,y}\\ \gamma_{xy}^{m}=u_{0,y}+v_{0,x},\;\;\gamma_{xy}^{f}=\theta_{x,y}+\theta_{y,x} \end{array}\right.$$

where the subscript ‘,’ signifies partial differentiation with respect to the associated coordinate, as illustrated by the example: $u_{0,x}=\partial u_{0}/\partial x$.

The *k*-th layer’s normal and shear stress distributions are defined as:

(7) $${\left\{\begin{array}{c} \sigma_{xx}\\ \sigma_{yy}\\ \tau_{xy} \end{array}\right\}}_{\left(k\right)}=\left[\begin{array}{ccc} Q_{11}^{\left(k\right)} & Q_{12}^{\left(k\right)} & 0\\ Q_{12}^{\left(k\right)} & Q_{22}^{\left(k\right)} & 0\\ 0 & 0 & Q_{66}^{\left(k\right)} \end{array}\right]{\left\{\begin{array}{c} \varepsilon_{xx}\\ \varepsilon_{yy}\\ \gamma_{xy} \end{array}\right\}}_{\left(k\right)},\;\;{\left\{\begin{array}{l} \tau_{xz}\\ \tau_{yz} \end{array}\right\}}_{\left(k\right)}=\left[\begin{array}{cc} Q_{55}^{\left(k\right)} & 0\\ 0 & Q_{44}^{\left(k\right)} \end{array}\right]{\left\{\begin{array}{c} \gamma_{xz}\\ \gamma_{yz} \end{array}\right\}}_{\left(k\right)}$$

in which the stiffness parameters are

(8) $$\left\{\begin{array}{lll} Q_{11}^{\left(k\right)}=\frac{E_{xx}^{\left(k\right)}}{1-\mu_{xy}^{\left(k\right)}\mu_{yx}^{\left(k\right)}}, & Q_{22}^{\left(k\right)}=\frac{E_{yy}^{\left(k\right)}}{1-\mu_{xy}^{\left(k\right)}\mu_{yx}^{\left(k\right)}}, & \;Q_{12}^{\left(k\right)}=\frac{\mu_{12}^{\left(k\right)}E_{11}^{\left(k\right)}}{1-\mu_{xy}^{\left(k\right)}\mu_{yx}^{\left(k\right)}}\\ Q_{44}^{\left(k\right)}=G_{yz}^{\left(k\right)}, & Q_{55}^{\left(k\right)}=G_{xz}^{\left(k\right)}, & Q_{66}^{\left(k\right)}=G_{zy}^{\left(k\right)} \end{array}\right.$$

where $E_{xx}^{\left(k\right)}$, $E_{yy}^{\left(k\right)}$, $\mu_{xy}^{\left(k\right)}$, $\mu_{yx}^{\left(k\right)}$, $G_{yz}^{\left(k\right)}$, $G_{xz}^{\left(k\right)}$, and $G_{zy}^{\left(k\right)}$ are the elastic constants.

Subsequently, the internal force, shear forces, and bending moment resultants are then derived by integrating stress distributions across the thickness of the *k*-th layer, as given by:

(9) $$\left\{\begin{array}{c} N_{xx}\\ N_{yy}\\ N_{xy}\\ M_{xx}\\ M_{yy}\\ M_{xy} \end{array}\right\}=\left[\begin{array}{cccccc} A_{11} & A_{12} & 0 & B_{11} & B_{12} & 0\\ A_{12} & A_{22} & 0 & B_{12} & B_{22} & 0\\ 0 & 0 & A_{66} & 0 & 0 & B_{66}\\ B_{11} & B_{12} & 0 & D_{11} & D_{12} & 0\\ B_{12} & B_{22} & 0 & D_{12} & D_{22} & 0\\ 0 & 0 & B_{66} & 0 & 0 & D_{66} \end{array}\right]\left\{\begin{array}{c} \varepsilon_{xx}^{m}\\ \varepsilon_{yy}^{m}\\ \gamma_{xy}^{m}\\ \varepsilon_{xx}^{f}\\ \varepsilon_{yy}^{f}\\ \gamma_{xy}^{f} \end{array}\right\}$$

where $A_{ij}$, $B_{ij}$, and $D_{ij}$ (i,j=1,2,6) represent the coupling bending stiffness, expressed as:

(10) $$A_{ij}=\sum_{k=1}^{3}Q_{ij}^{\left(k\right)}(z_{k}-z_{k-1}),\;B_{ij}=\frac{1}{2}\sum_{k=1}^{3}Q_{ij}^{\left(k\right)}(z_{k}^{2}-z_{k-1}^{2}),\;D_{ij}=\frac{1}{3}\sum_{k=1}^{3}Q_{ij}^{\left(k\right)}(z_{k}^{3}-z_{k-1}^{3})$$

For the composite sandwich plate, its top and bottom surfaces unsatisfied the shear stress-free boundary conditions. A shear-corrected expression [37] of the transverse shear force resultants is expressed as:

(11) $$\left\{\begin{array}{c} Q_{xz}\\ Q_{yz} \end{array}\right\}=\left[\begin{array}{cc} C_{55} & 0\\ 0 & C_{44} \end{array}\right]\left\{\begin{array}{c} \theta_{x}+w_{0,x}\\ \theta_{y}+w_{0,y} \end{array}\right\}$$

in which

(12) $$\frac{1}{C_{44}}=\sum_{k=1}^{3}\frac{9Z_{k}}{4Q_{44}^{\left(k\right)}}/H^{2},\;\;\frac{1}{C_{55}}=\sum_{k=1}^{3}\frac{9Z_{k}}{4Q_{55}^{\left(k\right)}}/H^{2}$$

(13) $$Z_{k}=z_{k}-z_{k-1}-\frac{8}{3}\left[{\left(z_{k}\right)}^{3}-{\left(z_{k-1}\right)}^{3}\right]/H^{2}+\frac{16}{5}\left[{\left(z_{k}\right)}^{5}-{\left(z_{k-1}\right)}^{5}\right]/H^{4}$$

##### The Orthogonal Ribs

The orthogonal ribs are also described by the FSDT of beams. According to the basic assumptions of FSDT, transverse normal stress is neglected, and the displacement components vary linearly through the thickness direction while maintaining continuity. Translational displacements and transverse rotations are assumed to be continuous at the conjunction lines of the first layer and the ribs. Perfect bonding without relative slip or separation is adopted between the ribs and the face sheets, ensuring displacement compatibility and force transmission at the interfaces. For the *j*-th rib oriented in a direction parallel to *x*-axis and the *i*-th rib oriented in a direction parallel to *y*-axis (*x* = *x_i_*), the displacement fields ($U_{b}$, $V_{b}$, $W_{b}$) are defined as:

(14) $$U_{b}(y_{j})=u_{b}(y_{j})+z_{b}\cdot \theta_{x}(y_{j}),\;\;W_{b}(y_{j})=w_{0}$$

(15) $$V_{b}(x_{i})=v_{b}(x_{i})+z_{b}\cdot \theta_{y}(x_{i}),\;\;W_{b}(x_{i})=w_{0}$$

where *u_b_* and *v_b_* are the displacements on the neutral axes of ribs at the x and y directions, and can be written as:

(16) $$u_{b}(y_{j})=u_{0}(y_{j})-e_{j}\cdot \theta_{x}(y_{j})$$

(17) $$v_{b}(x_{i})=v_{0}(x_{i})-e_{i}\cdot \theta_{y}(x_{i})$$

where *e_j_* = *h*_2_/2 + *h*_1_ + *h_bj_*/2 and *e_i_* = *h*_2_/2 + *h*_1_ + *h_bi_*/2

The corresponding strains are given as:

(18) $$\varepsilon_{bxx}(y_{j})=u_{0,x}(y_{j})-e_{j}\cdot \theta_{x,x}(y_{j})+z_{b}\cdot \theta_{x,x}(y_{j}),\;\;\gamma_{bxz}(y_{j})=\theta_{x}(y_{j})+w_{0,x}$$

(19) $$\varepsilon_{byy}(x_{i})=v_{0,y}(x_{i})-e_{i}\cdot \theta_{y,y}(x_{i})+z_{b}\cdot \theta_{y,y}(x_{i}),\;\;\gamma_{byz}(x_{i})=\theta_{y}(x_{i})+w_{0,y}$$

Then, the normal stress and shear stress are given as:

(20) $$\sigma_{bxx}(y_{j})=Q_{11}^{bj}\varepsilon_{bxx}(y_{j}),\;\;\tau_{bxz}(y_{j})=k_{s}Q_{55}^{bj}\gamma_{bxz}(y_{j})$$

(21) $$\sigma_{byy}(x_{i})=Q_{22}^{bi}\varepsilon_{byy}(x_{i}),\;\;\tau_{byz}(x_{i})=k_{s}Q_{44}^{bi}\gamma_{byz}(x_{i})$$

where the shear correction factor $k_{s}$ = 5/6. For symmetrical laminates with relatively low anisotropy, using 5/6 as the shear correction factor is a common practice in both engineering and academic research. It can ensure calculation accuracy while also considering solution efficiency, and it is consistent with the analysis conditions and structural characteristics of this paper.

Therefore, the stiffness parameters for the isotropic rib are expressed as:

(22) $$Q_{11}^{bj}=\frac{E_{b}}{1-\mu_{b}^{2}},\;\;Q_{55}^{bj}=\frac{E_{b}}{2(1-\mu_{b})},\;\;Q_{22}^{bi}=\frac{E_{b}}{1-\mu_{b}^{2}},\;\;Q_{44}^{bi}=\frac{E_{b}}{2(1-\mu_{b})}$$

Subsequently, the internal forces, bending moments, and shear forces of the rib-stiffened sandwich are determined as:

(23) $$N_{bxx}(y_{j})=A_{11}^{bj}\varepsilon_{bxx}^{m}(y_{j}),\;\;M_{bxx}(y_{j})=D_{11}^{bj}\varepsilon_{bxx}^{f}(y_{j}),\;\;Q_{bxz}(y_{j})=C_{55}^{bj}\gamma_{bxz}(y_{j})$$

(24) $$N_{byy}(x_{i})=A_{22}^{bi}\varepsilon_{byy}^{m}(x_{i}),\;\;M_{byy}(x_{i})=D_{22}^{bi}\varepsilon_{byy}^{f}(x_{i}),\;\;Q_{byz}(x_{i})=C_{44}^{bi}\gamma_{byz}(x_{i})$$

with

(25) $$\left\{\begin{array}{l} A_{11}^{bj}=Q_{11}^{bj}h_{bj},\;\;\;\;D_{11}^{bj}=Q_{11}^{bj}h_{bj}^{3}\;/12,\;\;\;\;C_{55}^{bj}=k_{s}Q_{55}^{bj}h_{bj}\\ \varepsilon_{bxx}^{m}(y_{j})=u_{0,x}(y_{j})-e_{j}\cdot \theta_{x,x}(y_{j}),\;\;\;\;\varepsilon_{bxx}^{f}(y_{j})=\theta_{x,x}(y_{j}) \end{array}\right.$$

(26) $$\left\{\begin{array}{l} A_{22}^{bi}=Q_{22}^{bi}h_{bi},\;\;\;\;D_{22}^{bi}=Q_{22}^{bi}h_{bi}^{3}\;/12,\;\;\;\;C_{44}^{bi}=k_{s}Q_{44}^{bi}h_{bi}\\ \varepsilon_{byy}^{m}(x_{i})=v_{0,y}(x_{i})-e_{i}\cdot \theta_{y,y}(x_{i}),\;\;\;\;\varepsilon_{byy}^{f}(x_{i})=\theta_{y,y}(x_{i}) \end{array}\right.$$

#### 2.3.2. Derivation of Motion Equations

In the dynamic analysis of the rib-stiffened sandwich, the equations of motion are derived based on Hamilton’s principle, and the strain energy as well as the kinetic energy of the structure are presented as:

(27) $$\begin{array}{ll} \int_{t_{1}}^{t_{2}}\delta U_{p}dt & =\int_{t_{1}}^{t_{2}}\int_{\Omega }\sum_{k=1}^{3}\int_{z_{k-1}}^{z_{k}}\left\{\sigma_{xx}\delta \varepsilon_{xx}+\sigma_{yy}\delta \varepsilon_{yy}+\tau_{xy}\delta \gamma_{xy}+\tau_{yz}\delta \gamma_{yz}+\tau_{xz}\delta \gamma_{xz}\right\}dzdxdydt\\ & =\int_{t_{1}}^{t_{2}}\int_{\Omega }\left\{\begin{array}{l} N_{xx}\delta \varepsilon_{xx}^{m}+N_{yy}\delta \varepsilon_{yy}^{m}+N_{xy}\delta \gamma_{xy}^{m}+M_{xx}\delta \varepsilon_{xx}^{f}\\ +M_{yy}\delta \varepsilon_{yy}^{f}+M_{xy}\delta \gamma_{xy}^{f}+Q_{xz}\delta \gamma_{xz}+Q_{yz}\delta \gamma_{yz} \end{array}\right\}dxdydt \end{array}$$

(28) $$\begin{array}{ll} \int_{t_{1}}^{t_{2}}\delta T_{p}dt & =\int_{t_{1}}^{t_{2}}\int_{\Omega }\sum_{k=1}^{3}\int_{z_{k-1}}^{z_{k}}\rho^{\left(k\right)}\left\{\dot{U}\delta \dot{U}+\dot{V}\delta \dot{V}+\dot{W}\delta \dot{W}\right\}dzdxdydt\\ & =-\int_{t_{1}}^{t_{2}}\int_{\Omega }\left\{\begin{array}{l} ({\bar{\rho }}_{0}{\ddot{u}}_{0}+{\bar{\rho }}_{1}{\ddot{\theta }}_{x})\delta u_{0}+({\bar{\rho }}_{1}{\ddot{u}}_{0}+{\bar{\rho }}_{2}{\ddot{\theta }}_{x})\delta \theta_{x}+\\ ({\bar{\rho }}_{0}{\ddot{v}}_{0}+{\bar{\rho }}_{1}{\ddot{\theta }}_{y})\delta v_{0}+({\bar{\rho }}_{1}{\ddot{v}}_{0}+{\bar{\rho }}_{2}{\ddot{\theta }}_{y})\delta \theta_{y}+{\bar{\rho }}_{0}{\ddot{w}}_{0}\delta w_{0} \end{array}\right\}dxdydt \end{array}$$

where $\left({\bar{\rho }}_{0},{\bar{\rho }}_{1},{\bar{\rho }}_{2}\right)=\sum_{k=1}^{3}\int_{z_{k-1}}^{z_{k}}\rho^{\left(k\right)}\cdot \left(1,z,z^{2}\right)dz$ and the overdot ‘$\dot{U}$’ denotes the differentiation with respect to time ‘t’; and $\rho^{\left(k\right)}$ is the mass density of the *k*-th layer.

The strain and kinetic energies of the $n_{1}$ ribs parallel to *x*-axis are expressed as:

(29) $$\begin{array}{ll} \int_{t_{1}}^{t_{2}}\delta U_{bx}dt & =\int_{t_{1}}^{t_{2}}\sum_{j=1}^{n_{1}}b_{j}\int_{-h_{bj}/2}^{h_{bj}/2}\int_{0}^{L_{x}}\left\{\sigma_{bxx}(y_{j})\delta \varepsilon_{bxx}(y_{j})+\tau_{bxz}(y_{j})\delta \gamma_{bxz}(y_{j})\right\}dxdzdt\\ & =\int_{t_{1}}^{t_{2}}\sum_{j=1}^{n_{1}}b_{j}\int_{\Omega }\left\{N_{bxx}\delta \varepsilon_{bxx}^{m}+M_{bxx}\delta \varepsilon_{bxx}^{f}+Q_{bxz}\delta \gamma_{bxz}\right\}\bar{\delta }(y-y_{j})dxdydt \end{array}$$

(30) $$\begin{array}{ll} \int_{t_{1}}^{t_{2}}\delta T_{bx}dt & =\int_{t_{1}}^{t_{2}}\sum_{j=1}^{n_{1}}b_{j}\int_{-h_{bj}/2}^{h_{bj}/2}\int_{0}^{L_{x}}\rho_{bj}\left\{{\dot{U}}_{b}(y_{j})\delta {\dot{U}}_{b}(y_{j})+{\dot{W}}_{b}\delta {\dot{W}}_{b}\right\}dxdzdt\\ & =-\int_{t_{1}}^{t_{2}}\sum_{j=1}^{n_{1}}b_{j}\int_{\Omega }\left\{\begin{array}{l} ({\bar{\rho }}_{x0}^{bj}{\ddot{u}}_{0}+{\bar{\rho }}_{x1}^{bj}{\ddot{\theta }}_{x})\delta u_{0}+\\ ({\bar{\rho }}_{x1}^{bj}{\ddot{u}}_{0}+{\bar{\rho }}_{x2}^{bj}{\ddot{\theta }}_{x})\delta \theta_{x}+{\bar{\rho }}_{x0}^{bj}{\ddot{w}}_{0}\delta w_{0} \end{array}\right\}\bar{\delta }(y-y_{j})dxdydt \end{array}$$

where $\left({\bar{\rho }}_{x0}^{bj},{\bar{\rho }}_{x1}^{bj},{\bar{\rho }}_{x2}^{bj}\right)=\int_{-h_{bj}/2}^{h_{bj}/2}\rho_{bj}\cdot \left(1,(z_{b}-e_{j}),{\left(z_{b}-e_{j}\right)}^{2}\right)dz_{b}$; $\bar{\delta }$ is the Dirac function; and $\rho_{bj}$ is the mass density of the *j*-th rib parallel to *x*-axis.

For the $n_{2}$ ribs parallel to *y*-axis, these are expressed as:

(31) $$\begin{array}{ll} \int_{t_{1}}^{t_{2}}\delta U_{by}dt & =\int_{t_{1}}^{t_{2}}\sum_{i=1}^{n_{2}}b_{i}\int_{-h_{bi}/2}^{h_{bi}/2}\int_{0}^{L_{y}}\left\{\sigma_{byy}(x_{i})\delta \varepsilon_{byy}(x_{i})+\tau_{byz}(x_{i})\delta \gamma_{byz}(x_{i})\right\}dydzdt\\ & =\int_{t_{1}}^{t_{2}}\sum_{i=1}^{n_{2}}b_{i}\int_{\Omega }\left\{N_{byy}\delta \varepsilon_{byy}^{m}+M_{byy}\delta \varepsilon_{byy}^{f}+Q_{byz}\delta \gamma_{byz}\right\}\bar{\delta }(x-x_{i})dxdydt \end{array}$$

(32) $$\begin{array}{ll} \int_{t_{1}}^{t_{2}}\delta T_{by}dt & =\int_{t_{1}}^{t_{2}}\sum_{i=1}^{n_{2}}b_{i}\int_{-h_{bi}/2}^{h_{bi}/2}\int_{0}^{L_{y}}\rho_{bi}\left\{{\dot{V}}_{b}(x_{i})\delta {\dot{V}}_{b}(x_{i})+{\dot{W}}_{b}\delta {\dot{W}}_{b}\right\}dydzdt\\ & =-\int_{t_{1}}^{t_{2}}\sum_{i=1}^{n_{2}}b_{i}\int_{\Omega }\left\{\begin{array}{l} ({\bar{\rho }}_{y0}^{bi}{\ddot{v}}_{0}+{\bar{\rho }}_{y1}^{bi}{\ddot{\theta }}_{y})\delta v_{0}+\\ ({\bar{\rho }}_{y1}^{bi}{\ddot{v}}_{0}+{\bar{\rho }}_{y2}^{bi}{\ddot{\theta }}_{y})\delta \theta_{y}+{\bar{\rho }}_{y0}^{bi}{\ddot{w}}_{0}\delta w_{0} \end{array}\right\}\bar{\delta }(x-x_{i})dxdydt \end{array}$$

where $\left({\bar{\rho }}_{y0}^{bi},{\bar{\rho }}_{y1}^{bi},{\bar{\rho }}_{y2}^{bi}\right)=\int_{-h_{bi}/2}^{h_{bi}/2}\rho_{bi}\cdot \left(1,(z_{b}-e_{i}),{\left(z_{b}-e_{i}\right)}^{2}\right)dz_{b}$; and $\rho_{bi}$ is the mass density of the *i*-th rib parallel to *y*-axis.

When the rib-stiffened sandwich plate is subjected to a normal excitation load $q_{z}(x,y)$ within the fluid domain, the external work is expressed as:

(33) $$\int_{t_{1}}^{t_{2}}\delta Wdt=\int_{t_{1}}^{t_{2}}\int_{\Omega }\left[q_{z}(x,y)+\bar{p}(x,y)\right]\delta w_{0}dxdydt$$

where $\bar{p}(x,y)$ represents the pressure jump induced by the transverse structural vibration.

The equation of Hamilton’s principle for the presented rib-stiffened sandwich is as follows:

(34) $$\int_{t_{1}}^{t_{2}}\left[\delta (T_{p}+T_{bx}+T_{by})-\delta (U_{p}+U_{bx}+U_{by})+\delta W\right]dt=0$$

Then the motion equations are derived as:

(35) $$\delta u_{0}:N_{x,x}+N_{xy,y}+\sum_{j=1}^{n_{1}}b_{j}N_{bxx,x}\bar{\delta }(y-y_{j})-\left({\bar{\rho }}_{0}{\ddot{u}}_{0}+{\bar{\rho }}_{1}{\ddot{\theta }}_{x}\right)-\sum_{j=1}^{n_{1}}b_{j}\left({\bar{\rho }}_{x0}^{bj}{\ddot{u}}_{0}+{\bar{\rho }}_{x1}^{bj}{\ddot{\theta }}_{x}\right)\bar{\delta }(y-y_{j})\;\;=0$$

(36) $$\delta v_{0}:N_{y,y}+N_{xy,x}+\sum_{i=1}^{n_{2}}b_{i}N_{byy,y}\bar{\delta }(x-x_{i})-\left({\bar{\rho }}_{0}{\ddot{v}}_{0}+{\bar{\rho }}_{1}{\ddot{\theta }}_{y}\right)-\sum_{i=1}^{n_{2}}b_{i}\left({\bar{\rho }}_{y0}^{bi}{\ddot{v}}_{0}+{\bar{\rho }}_{y1}^{bi}{\ddot{\theta }}_{y}\right)\;\bar{\delta }(x-x_{i})=0$$

(37) $$\begin{array}{ll} \delta \theta_{x}: & M_{x,x}+M_{xy,y}+\sum_{j=1}^{n_{1}}b_{j}(M_{bxx,x}-e_{j}N_{bxx,x}-Q_{bxz})\bar{\delta }(y-y_{j})-Q_{xz}-({\bar{\rho }}_{1}{\ddot{u}}_{0}+{\bar{\rho }}_{2}{\ddot{\theta }}_{x})\\ & -\sum_{j=1}^{n_{1}}b_{j}\left({\bar{\rho }}_{x1}^{bj}{\ddot{u}}_{0}+{\bar{\rho }}_{x2}^{bj}{\ddot{\theta }}_{x}\right)\bar{\delta }(y-y_{j})\;\;=0 \end{array}$$

(38) $$\begin{array}{ll} \delta \theta_{y}: & M_{y,y}+M_{xy,x}++\sum_{i=1}^{n_{2}}b_{i}\left(M_{byy,y}-e_{i}N_{byy,y}-Q_{byz}\right)\bar{\delta }(x-x_{i})-Q_{yz}-({\bar{\rho }}_{1}{\ddot{v}}_{0}+{\bar{\rho }}_{2}{\ddot{\theta }}_{y})\\ & -\sum_{i=1}^{n_{2}}b_{i}\left({\bar{\rho }}_{y1}^{bi}{\ddot{v}}_{0}+{\bar{\rho }}_{y2}^{bi}{\ddot{\theta }}_{y}\right)\bar{\delta }(x-x_{i})=0 \end{array}$$

(39) $$\begin{array}{ll} \delta w_{0}: & Q_{xz,x}+Q_{yz,y}-{\bar{\rho }}_{0}{\ddot{w}}_{0}+q_{z}+\bar{p}+\sum_{j=1}^{n_{1}}b_{j}(Q_{bxz,x}-{\bar{\rho }}_{x0}^{bj}{\ddot{w}}_{0})\bar{\delta }(y-y_{j})\\ & +\sum_{i=1}^{n_{2}}b_{i}(Q_{byz,y}-{\bar{\rho }}_{y0}^{bi}{\ddot{w}}_{0})\bar{\delta }(x-x_{i})=0 \end{array}$$

#### 2.3.3. Fluid–Solid Coupling and Acoustic Pressure Distribution

Equation (39) describes the lateral vibration response of the rib-stiffened sandwich under the action of fluid–solid coupling. The acoustic pressure distribution at the spatial field point satisfies the Helmholtz equation, and its expression is as follows:

(40) $$\frac{\partial^{2}p}{\partial x^{2}}+\frac{\partial^{2}p}{\partial y^{2}}+\frac{\partial^{2}p}{\partial z^{2}}+\frac{\omega^{2}}{c_{0}^{2}}p=0$$

where $c_{0}$ is the acoustic velocity in fluid; ω is the circular frequency; and the acoustic wave number is $k_{0}=\omega /c_{0}$.

Ref. [38] has presented an expression for the acoustic pressure associated with a thin plate as follows:

(41) $$p(M_{0})=\int_{S_{p}}\bar{p}(x,y)\frac{\partial G(M,M_{0})}{\partial z_{M}}dS_{p}$$

where $S_{p}$ is defined as the plate surface; M(x,y,z) is an arbitrary point on $S_{p}$; $z_{M}$ denotes the normal direction to the surface at M; and the Green’s function $G(M,M_{0})$ is expanded in the wavenumber domain, as given by:

(42) $$G(M,M_{0})=\int_{-\infty }^{+\infty }\int_{-\infty }^{+\infty }\frac{j_{1}e^{jk_{x}(x-x_{0})}e^{jk_{y}(y-y_{0})}e^{jk_{z}\left|z-z_{0}\right|}}{8\pi^{2}k_{z}}dk_{x}\;dk_{y}$$

with

(43) $$k_{z}=\left\{\begin{array}{c} \sqrt{k_{0}^{2}-k_{x}^{2}-k_{y}^{2}},\;\;k_{0}^{2}\geq k_{x}^{2}+k_{y}^{2}\\ j_{1}\sqrt{k_{x}^{2}+k_{y}^{2}-k_{0}^{2}},\;\;k_{0}^{2}<k_{x}^{2}+k_{y}^{2} \end{array}\right.$$

where $j_{1}$ is the imaginary unit; and $k_{x}$ and $k_{y}$ are the Fourier’s variables.

The Green’s function $G(M,M_{0})$ in the wavenumber domain is strictly valid for all r > 0. In the near-field (r≤O(λ)), its full form must be retained to capture evanescent waves for accurate fluid–solid coupling, while in the far-field ($k_{0}r\gg 1$), the evanescent component decays, allowing for simplified radiation noise calculations under the Sommerfeld condition.

The continuity condition of normal acceleration at the fluid–structure interface is derived from the Euler equation of fluid dynamics, which governs the motion of inviscid fluids. For small-amplitude acoustic perturbations, the convective acceleration term υ⋅∇υ is negligible, and the linearized Euler equation in the normal direction to the interface is as follows:

(44) $$\rho_{0}\frac{\partial \upsilon_{n}}{\partial t}=-\frac{\partial p}{\partial n}$$

where $\rho_{0}$ is the fluid density; $\upsilon_{n}$ is the normal component of the fluid velocity; t is time; p is the acoustic pressure; and n is the unit normal vector to the interface. At the fluid–solid interface, the kinematic continuity condition requires that the normal velocity of the fluid equals the normal velocity of the solid structure:

(45) $$\upsilon_{n}=\frac{\partial w}{\partial t}$$

where w is the normal displacement of the solid. Substituting this into the linearized Euler equation yields:

(46) $$\rho_{0}\frac{\partial^{2}w}{\partial t^{2}}=-\frac{\partial p}{\partial n}$$

where, in the frequency domain, the time derivative ∂/∂t is replaced by jω; while ω is the angular frequency. The normal acceleration of the solid is then $\partial^{2}w/\partial t^{2}=-\omega^{2}w$, leading to the frequency-domain form of the continuity condition:

(47) $$\rho_{0}\omega^{2}w=\frac{\partial p}{\partial n}$$

By invoking this condition, the continuity of normal acceleration at the fluid–structure interface is expressed as:

(48) $$\rho_{0}\omega^{2}w_{0}(x_{0},y_{0})={\left.\frac{\partial p(M_{0})}{\partial z_{0}}\right|}_{z_{0}=0}=\int_{S_{p}}\bar{p}(x,y)\frac{\partial^{2}G(M,M_{0})}{\partial z_{0}\partial z_{M}}dS_{p}$$

Treating the current rib-stiffened sandwich structure as a thin plate and substituting Equation (39) into Equation (48), the transverse vibration equation is reformulated as:

(49) $$\delta w_{0}:\;-\int_{\Omega }\left(\begin{array}{l} Q_{xz,x}+Q_{yz,y}-{\bar{\rho }}_{0}{\ddot{w}}_{0}+q_{z}+\\ \sum_{j=1}^{n_{1}}b_{j}(Q_{bxz,x}-{\bar{\rho }}_{x0}^{bj}{\ddot{w}}_{0})\bar{\delta }(y-y_{j})\\ +\sum_{i=1}^{n_{2}}b_{i}(Q_{byz,y}-{\bar{\rho }}_{y0}^{bi}{\ddot{w}}_{0})\bar{\delta }(x-x_{i}) \end{array}\right){\left.\frac{\partial^{2}G}{\partial z_{M}\partial z_{0}}\right|}_{z_{M},z_{0}=0}dxdy=\rho_{0}\omega^{2}w_{0}$$

Thus, Equations (35)–(38) and (49) constitute the new motion equations. Subsequently, by leveraging the derived internal force and moment resultants, the equations are expressed in five unknown variables ($u_{0}$, $v_{0}$, $\theta_{x}$, $\theta_{y}$, $w_{0}$), as listed in Appendix A. In addition, the equations can also degenerate into the vacuum case when $\rho_{0}=0$.

### 2.4. Analytical Solution

For the rib-stiffened sandwich under the four-sided simply supported boundary conditions, the displacement field is expanded in the form of a double trigonometric series to meet the displacement and rotation constraint requirements of the simply supported boundaries:

(50) $$\left\{\begin{array}{c} \left(u_{0},\theta_{x}\right)\\ \left(v_{0},\theta_{y}\right)\\ w_{0} \end{array}\right\}=e^{j_{1}\omega t}\sum_{m,n}\left\{\begin{array}{c} (u_{0mn},\theta_{xmn})\psi_{x}\varphi_{y}\\ (v_{0mn},\theta_{ymn})\varphi_{x}\psi_{y}\\ w_{0mn}\varphi_{x}\varphi_{y} \end{array}\right\}$$

where $\sum_{m,n}$ denotes $\sum_{m=1}^{\infty }\sum_{n=1}^{\infty }$; m and n denote the half-wave numbers; and $\varphi_{x}=\sin\alpha x$, $\varphi_{y}=\sin\beta y$, $\psi_{x}=\cos\alpha x$, $\psi_{y}=\cos\beta y$. $u_{0mn}$, $v_{0mn}$, $w_{0mn}$, $\theta_{xmn}$, and $\theta_{ymn}$ are the modal amplitudes of the displacement and rotation components, with the wavenumbers given by $\alpha =m\pi /L_{x}$ and $\beta =n\pi /L_{y}$.

The external force is written as:

(51) $$q_{z}=e^{j_{1}\omega t}\sum_{m,n}(q_{zmn}\varphi_{x}\varphi_{y})$$

where $q_{zmn}=\int_{0}^{L_{x}}\int_{0}^{L_{y}}(4q_{z}\varphi_{x}\varphi_{y}/L_{x}L_{y})dxdy$.

#### 2.4.1. Free Vibration

For the free vibration case, the external excitation is specified as $q_{z}$ = 0. By employing the Navier solution procedure, the five governing equations of motion are projected onto the orthogonal modal shapes, which include $\cos(p\pi x/L_{x})\sin(q\pi y/L_{y})$, $\sin(p\pi x/L_{x})\cos(q\pi y/L_{y})$, and $\sin(p\pi x_{0}/L_{x})\sin(q\pi y_{0}/L_{y})$, where p,q=1,2…+∞. To extract the vibration mode (*m*, *n*), we set p=m and q=n, leading to the free vibration equations for this mode:

(52) $$\left[\begin{array}{ccccc} {\bar{R}}_{11} & {\bar{R}}_{12} & {\bar{R}}_{13} & {\bar{R}}_{14} & {\bar{R}}_{15}\\ {\bar{R}}_{21} & {\bar{R}}_{22} & {\bar{R}}_{23} & {\bar{R}}_{24} & {\bar{R}}_{25}\\ {\bar{R}}_{31} & {\bar{R}}_{32} & {\bar{R}}_{33} & {\bar{R}}_{34} & {\bar{R}}_{35}\\ {\bar{R}}_{41} & {\bar{R}}_{42} & {\bar{R}}_{43} & {\bar{R}}_{44} & {\bar{R}}_{45}\\ {\bar{R}}_{51} & {\bar{R}}_{52} & {\bar{R}}_{53} & {\bar{R}}_{54} & {\bar{R}}_{55} \end{array}\right]\left\{\begin{array}{c} u_{0mn}\\ v_{0mn}\\ \theta_{xmn}\\ \theta_{ymn}\\ w_{0mn} \end{array}\right\}=\left\{\begin{array}{c} 0\\ 0\\ 0\\ 0\\ 0 \end{array}\right\}$$

where the coefficient matrices ${\bar{R}}_{ij}\;(i,j=1,2,\ldots ,5)$ are provided in detail in Appendix B. By setting the determinant of the system coefficient matrix to 0, the natural frequency corresponding to the vibration mode (*m*, *n*) can be solved explicitly.

#### 2.4.2. Forced Vibration

For the forced vibration analysis, the external excitation is non-zero. To obtain the vibration response using the Navier solution approach, a modal truncation is introduced where m,p=1,2,3…M and n,q=1,2,3…N. This truncation procedure leads to the transformed forced vibration equations:

(53) $${\left[\begin{array}{ccccc} \left[{\bar{S}}_{11}\right] & \left[{\bar{S}}_{12}\right] & \left[{\bar{S}}_{13}\right] & \left[{\bar{S}}_{14}\right] & \left[{\bar{S}}_{15}\right]\\ \left[{\bar{S}}_{21}\right] & \left[{\bar{S}}_{22}\right] & \left[{\bar{S}}_{23}\right] & \left[{\bar{S}}_{24}\right] & \left[{\bar{S}}_{25}\right]\\ \left[{\bar{S}}_{31}\right] & \left[{\bar{S}}_{32}\right] & \left[{\bar{S}}_{33}\right] & \left[{\bar{S}}_{34}\right] & \left[{\bar{S}}_{35}\right]\\ \left[{\bar{S}}_{41}\right] & \left[{\bar{S}}_{42}\right] & \left[{\bar{S}}_{43}\right] & \left[{\bar{S}}_{44}\right] & \left[{\bar{S}}_{45}\right]\\ \left[{\bar{S}}_{51}\right] & \left[{\bar{S}}_{52}\right] & \left[{\bar{S}}_{53}\right] & \left[{\bar{S}}_{54}\right] & \left[{\bar{S}}_{55}\right] \end{array}\right]}_{5MN\times 5MN}\left\{\begin{array}{c} {\left[{\bar{U}}_{1}\right]}_{MN\times 1}\\ {\left[{\bar{U}}_{2}\right]}_{MN\times 1}\\ {\left[{\bar{U}}_{3}\right]}_{MN\times 1}\\ {\left[{\bar{U}}_{4}\right]}_{MN\times 1}\\ {\left[{\bar{U}}_{5}\right]}_{MN\times 1} \end{array}\right\}=\left\{\begin{array}{c} {\left[0\right]}_{MN\times 1}\\ {\left[0\right]}_{MN\times 1}\\ {\left[0\right]}_{MN\times 1}\\ {\left[0\right]}_{MN\times 1}\\ {\left[\bar{F}\right]}_{MN\times 1} \end{array}\right\}$$

with

(54) $$\left\{\begin{array}{l} {\left[{\bar{U}}_{1}\right]}_{MN\times 1}={\left[u_{011},u_{012},\ldots ,u_{01N},u_{021},u_{022},\ldots ,u_{02N},\ldots ,u_{0MN}\right]}^{T}\\ {\left[{\bar{U}}_{2}\right]}_{MN\times 1}={\left[v_{011},v_{012},\ldots ,v_{01N},v_{021},v_{022},\ldots ,v_{02N},\ldots ,v_{0MN}\right]}^{T}\\ {\left[{\bar{U}}_{3}\right]}_{MN\times 1}={\left[\theta_{x11},\theta_{x12},\ldots ,\theta_{x1N},\theta_{x21},\theta_{x22},\ldots ,\theta_{x2N},\ldots ,\theta_{xMN}\right]}^{T}\\ {\left[{\bar{U}}_{4}\right]}_{MN\times 1}={\left[\theta_{y11},\theta_{y12},\ldots ,\theta_{y1N},\theta_{y21},\theta_{y22},\ldots ,\theta_{y2N},\ldots ,\theta_{yMN}\right]}^{T}\\ {\left[{\bar{U}}_{5}\right]}_{MN\times 1}={\left[w_{011},w_{012},\ldots ,w_{01N},w_{021},w_{022},\ldots ,w_{02N},\ldots ,w_{0MN}\right]}^{T} \end{array}\right.$$

(55) $${\left[\bar{F}\right]}_{MN\times 1}={\left[{\bar{O}}_{11},{\bar{O}}_{12},\ldots {\bar{O}}_{1N},{\bar{O}}_{21},{\bar{O}}_{22},\ldots {\bar{O}}_{2N},\ldots {\bar{O}}_{pq},\ldots {\bar{O}}_{MN}\right]}^{T}$$

where $\left[{\bar{S}}_{ij}\right]={\left[{\bar{T}}_{ij}(pN-N+q,mN-N+n)\right]}_{MN\times MN}$ and ${\bar{O}}_{pq}$, and are listed in Appendix C.

Once the displacement responses are derived, the normal mean square velocity (MQV) level is defined as:

(56) $$\mathrm{MQV}=10\mathrm{lg}\left(\frac{1}{2L_{x}L_{y}}\int_{0}^{L_{x}}\int_{0}^{L_{y}}{\left|v_{z}\right|}^{2}dxdy/{\mathrm{ref}}_{m}\right)$$

Here, $v_{z}=2j\pi fw_{0}$ denotes the vibration velocity in the z-direction, where the excitation frequency is defined as f=ω/2π and the reference level for MQV is ${\mathrm{ref}}_{m}=1\times 10^{-18}\;m^{2}s^{-2}$. The sound pressure distribution is obtained from Equation (41), with a reference sound pressure level of 1 × 10^−6^ Pa.

## 3. Validation of Method Effectiveness

Free and forced vibration investigations of the orthogonal rib-stiffened PVC foam sandwich structures with reinforcing and weakening phases in vacuum and water are conducted in this section, where the acoustic velocity and mass density of water are $\rho_{0}=1000\;{\mathrm{kg}/m}^{3}$ and $c_{0}=1500\;m/s$.

### 3.1. Simulation Verification Description

The orthogonal rib-stiffened PVC foam sandwich structures with reinforcing and weakening phases are further considered. For the geometric parameters of the structure, the plate length and width are specified as $L_{x}=1.26\;m$ and $L_{y}=0.9\;m$. The thicknesses of the lower face sheet, core layer, and upper face sheet are defined as $h_{1}=0.005\;m$, $h_{2}=0.04\;m$, and $h_{3}=0.005\;m$. Additionally, the spacing between the adjacent reinforcements, the reinforcement diameter, and the cavity diameter are given as l=0.06 m, $d_{r}=0.01\;m$, and $d_{c}=0.02\;m$. The height and width of the rectangular rib is 0.03 m and 0.01 m. The face sheets comprise glass fiber-reinforced plastics (GFRP), with its Young’s modulus as *E*_11_ = *E*_22_ = 23.5 GPa and *E*_33_ = 4.5 GPa; shear modulus as *G*_12_ = *G*_13_ = *G*_23_ = 3.23 GPa; a Poisson’s ratio of $\mu_{12}=\mu_{13}=\mu_{23}=0.14$; and a mass density of 1800 kg/m^3^. The rib stiffeners comprise polyurethane (PU), with the Young’s modulus as *E_b_* = 14 GPa; a Poisson’s ratio of $\mu_{b}=0.28$; and a mass density of 760 kg/m^3^. The core material is PVC-H320, with its Young’s modulus as *E_cm_* = 0.45 GPa; a Poisson’s ratio of $\mu_{cm}=0.30$; and a mass density of 320 kg/m^3^. The reinforcement has the Young’s modulus as *E_re_* = 55 GPa; a Poisson’s ratio of $\mu_{re}=0.30$; and mass density of 1300 kg/m^3^. The weak modulus of the cavity is assumed as 1Pa. The theoretically predicted vibration results under vacuum and when submerged in water are validated by comparisons with the numerical simulation outcomes, including FE analyses conducted in ANSYS 16.0 and coupled FE/BE analyses performed in Virtual.Lab 13.3. Three cases of orthogonal ribs are taken into account as: (1) *n*_1_ = 1 and *n*_2_ = 2 (*y*_1_ = 0.45 m, *x*_1_ = 0.42 m, *x*_2_ = 0.84 m); (2) *n*_1_ = 2 and *n*_2_ = 2 (*y*_1_ = 0.30 m, *y*_2_ = 0.60 m, *x*_1_ = 0.42 m, *x*_2_ = 0.84 m); and (3) *n*_1_ = 2 and *n*_2_ = 3 (*y*_1_ = 0.30 m, *y*_2_ = 0.60 m, *x*_1_ = 0.30 m, *x*_2_ = 0.63 m, *x*_3_ = 0.96 m).

**Figure 3:**
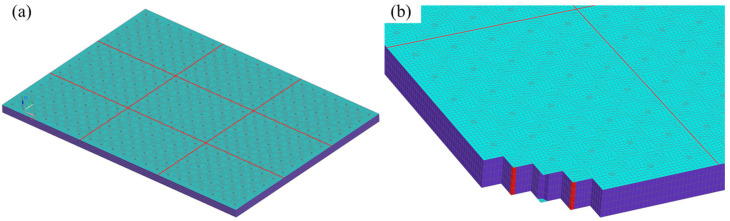
The FE model of orthogonal rib-stiffened PVC foam sandwich structures with reinforcing and weakening phases: (**a**) the whole structure; (**b**) the partial section.

To demonstrate the numerical analysis procedure, a rib-stiffened sandwich structure with *n*_1_ = 2 and *n*_2_ = 2 is selected as a case study, and its FE implementation in ANSYS is detailed as follows: a convergent FE model is established through iterative mesh refinement, consisting of 238,152 elements with a minimum mesh size of 2.5 mm (as illustrated in Figure 3), where the face sheets, core layer (including both the core material and internal reinforcements), and ribs are assigned SHELL181, SOLID185 and BEAM188 elements respectively, and the rib-stiffened structure is subjected to simply supported boundary conditions.

(57) $$\begin{array}{l} v=0,\;w=0\;\;\;\mathrm{at}\;\;x=0,\;L_{x}\\ u=0,\;w=0\;\;\;\mathrm{at}\;\;y=0,\;L_{y} \end{array}$$

**Figure 4:**
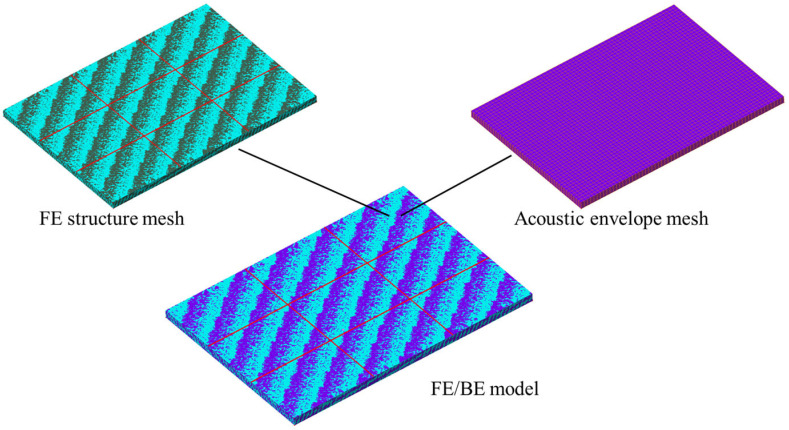
The mesh mapping and FE/BE mesh in Virtual.Lab.

FE analyses are first performed to investigate the free vibration of the rib-stiffened sandwich structure in a vacuum environment. Using the obtained dry-mode vibration characteristics, coupled FE/BE analyses are subsequently carried out in Virtual.Lab to predict the vibration response of the structure submerged in water. To establish the acoustic coupling, the structural FE mesh is enclosed within an acoustic envelope mesh, and the FE/BE model is constructed via mesh mapping, as depicted in Figure 4. This acoustic envelope mesh comprises 7420 shell elements and is validated to ensure the convergence of FE/BE analyses, with the direct BE method adopted for acoustic field calculations.

### 3.2. Convergence Verification

Before performing the simulation calculation and after the two steps of the Mori–Tanaka homogenization method, the equivalent elastic constants for each stage are shown in the following Table 1.

**Figure 5:**
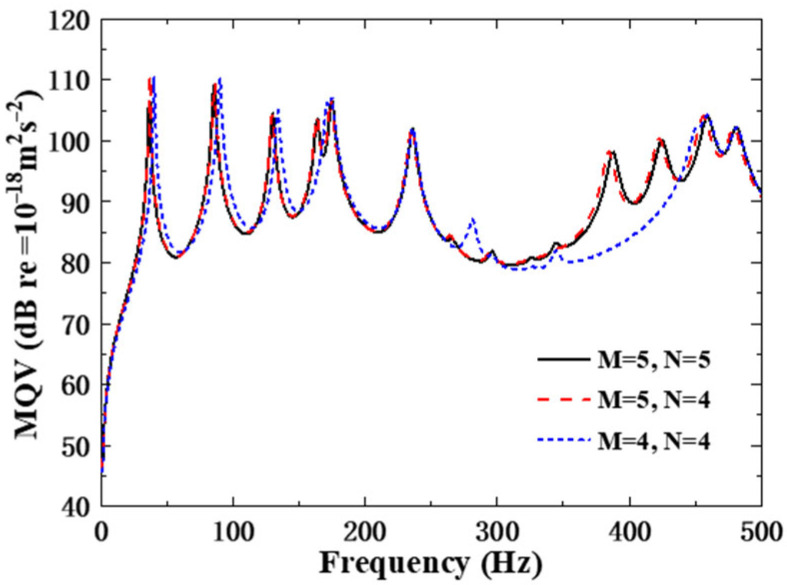
Convergence of natural frequencies versus truncation orders M and N.

In order to analyze the relationship between the truncation orders (M, N) and the natural frequencies, convergence analysis was performed by gradually increasing the values of M and N. The calculation results show that the natural frequency gradually converges with the increase in truncation orders, and when M and N reach a certain threshold, the natural frequency value tends to be stable and no longer changes significantly, as clearly demonstrated by the convergence curves in the Figure 5.

On this basis, the natural frequencies of the underwater (*m*, *n*) modes of the composite structure are listed in the following Table 2 (*m* = 1, 2, 3, 4, 5, 6, and *n* = 1, 2, 3, 4, 5), which complements the graphical results and provides specific data supporting the convergence analysis. From the table, it can be seen that the natural frequencies of modes (6, *n*) and (*m*, 5) are both greater than 500 Hz, which is consistent with the high-frequency distribution law reflected in the Figure 5. Therefore, by choosing M = 5 and N = 4 as the truncation values, we can ensure that all the modes that contribute to the response within the analysis frequency range are included without wasting computing resources due to the inclusion of too many non-essential higher-order modes—this is the optimal choice that balances accuracy and efficiency.

To ensure the reliability of the numerical results and to eliminate the influence of mesh discretization error, a mesh convergence study was performed in ANSYS. As shown in Table 3, two finite element mesh schemes were established: an original mesh with 238,152 elements and a refined mesh with 365,640 elements. The results showed that the relative differences in the natural frequencies of all considered modes between the refined and original meshes were less than 0.6%. This level of discrepancy satisfied the accuracy requirements for both engineering and academic analyses, confirming that the original mesh scheme has achieved mesh convergence and is sufficiently refined to yield reliable results for the subsequent vibro-acoustic response investigations.

In addition, the influence of acoustic grid density on the calculation results was verified, and the calculation results of each mode under the two grid schemes (7420 and 11,280) were compared, as shown in Table 4. The results show that under the calculated modes, the calculation differences in the two grids are all less than 0.5%, which proves that when the number of acoustic grids reaches 7420, the numerical solution is close to the grid-independent solution. Further increasing the grid density has a limited effect on improving the accuracy; therefore, in this study, 7420 is selected as the benchmark density for the acoustic grid, which can ensure calculation accuracy and improve calculation efficiency.

### 3.3. Validity for Free Vibration

Table 5 displays the comparison of achieved analytical and numerical natural frequencies, both in vacuum and in water. The first five modes of presented sandwich structures with three cases of orthogonal ribs are listed, and the errors of the analytical results are calculated. It can be seen that there is good consistency between the first five analytical and numerical natural frequencies, while all the errors were no more than 3.87%. Hence, the correctness of the proposed analytical method is confirmed for the free vibration analysis of the rib-stiffened sandwich structure. In addition, when the number of ribs increases and the cases of ribs tend to be concentrated, all of the first five natural frequencies show rising trends, except for the mode (1, 2) of rib case (*n*_1_ = 2, *n*_2_ = 2) and the mode (3, 1) of rib case (*n*_1_ = 2, *n*_2_ = 3)—in general, the ribs enhance the global bending stiffness of whole sandwich structure.

Under the three different rib arrangement conditions, the natural frequencies in the water environment are significantly lower than those in the vacuum environment. The frequency ratio $f_{v}/f_{w}$ is greater than 2.9 in all cases. This clearly indicates that the additional mass effect of the water significantly reduces the natural frequencies of the structure. At the same time, the frequency ratio decreases as the modal order increases; for example, in the (*n*_1_ = 1, *n*_2_ = 2) condition, from the mode (1, 1) of 4.25 to the mode (2, 2) of 2.99, it shows that the influence of the additional mass on higher-order modes is relatively small. Moreover, the influence of different rib arrangements on the frequency ratio is small, indicating that the additional mass effect is mainly dominated by the coupling effect between the fluid and the structure, rather than the stiffness change in the ribs themselves.

### 3.4. Validity for Forced Vibration

**Figure 6:**
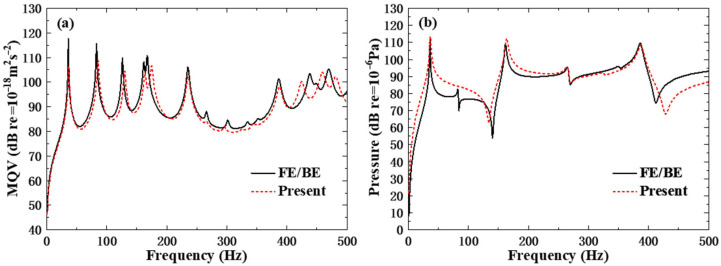
Comparison of the ESL theoretical modeling approach with the results of the FE/BE numerical approach: (**a**) MQV; (**b**) field point sound pressure.

Calculating the underwater forced vibration response of orthogonal rib-stiffened PVC foam sandwich structures with reinforcing and weakening phases based on the second reinforcement rib case (*n*_1_ = 2, *n*_2_ = 2). A concentrated force in the z-direction is applied to the surface of the reinforced plate. The point of application of the excitation force is at (0.3, 0.3, 0), and the magnitude of the force is 1 N. The frequency range of the analysis is 1–500 Hz, with a step size of 1 Hz. In the underwater acoustic analysis, the fluid domain is assumed to be infinite, homogeneous, compressible, inviscid, and quiescent water, which satisfies the Helmholtz equation in the frequency domain. For the sound pressure evaluation, the coordinate of the field point in the sound pressure is (0.63, 0.45, 5), which is located in the far field to characterize the underwater acoustic radiation characteristics of the structure. The results of the ESL theoretical modeling approach based on core equivalent homogenization are compared with the results of the FE/BE numerical simulation approach, as shown in Figure 6. As can be seen, the analytical solution of the ESL theoretical modeling approach agrees well with the numerical solution. In the region of low frequencies, the MQV curves essentially overlap, but the peaks of the curves are shifted in the region of high frequencies. For the field point sound pressure, there are good agreements between the present analytical results and the ones from FE/BE simulation. Thus, the present FSDT-based analytical approach has good accuracy for the underwater acoustic vibration research of rib-stiffened PVC foam sandwich structures with reinforcing and weakening phases.

**Figure 7:**
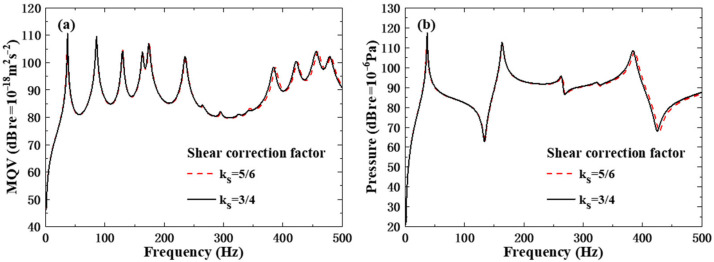
Influence of shear correction factors: (**a**) MQV; (**b**) field point sound pressure.

Figure 7 compares the influence of different shear correction factors on the calculated vibro-acoustic results. Parametric studies confirm that the shear correction factor ($k_{s}$) exhibits a frequency-dependent impact on vibro-acoustic responses. At low frequencies, the influence of $k_{s}$ on both the MQV and field point sound pressure is negligible with the curves for different values nearly coinciding. Nevertheless, in the high-frequency domain (>200 Hz), a larger $k_{s}$ value significantly amplifies the vibration response, inducing distinct deviations in the radiated sound pressure. These results emphasize that while the low-frequency analysis can tolerate a certain flexibility in selecting α, a rational and physically justified choice of $k_{s}$ is critical for high-frequency simulations to guarantee reliable predictions of structural vibration and noise.

## 4. Acoustic Vibration Performance

The underwater acoustic vibration radiation performance of orthogonal rib-stiffened PVC foam sandwich structures with reinforcing and weakening phases is analyzed by varying the material and geometrical parameters based on the rib case (*n*_1_ = 2, *n*_2_ = 2).

### 4.1. Influence of Core Layer Parameters

#### 4.1.1. Effect of Core Layer Materials

To explore the influence of core layer materials on the acoustic vibration performances of the orthogonal rib-stiffened PVC foam sandwich structures with reinforcing and weakening phases, three cases of PVC foam are respectively adopted as core layer materials, as listed in Table 6. The underwater natural frequencies of the sandwich are given in Table 7. It can be observed that across PVC-H100, PVC-H250, and PVC-H320, the Young’s modulus and mass density of the core layer both present a gradually increasing tendency. All the natural frequencies increase significantly, indicating that the stiffness of PVC foam core exhibits greater effects on the inherent vibrations. The underlying mechanism is that the added mass of water dominates the mode mass of the underwater, and thus the increase in the mass density of the core material has little influence.

**Figure 8:**
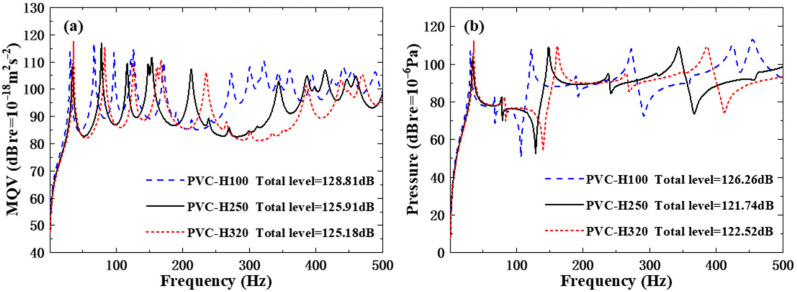
Underwater forced vibration response of sandwich plates with different core layer materials: (**a**) MQV; (**b**) field point sound pressure.

Figure 8 presents the MQV and field point sound pressure of the sandwich with different core layer material. Across PVC-H100, PVC-H250, and PVC-H320, both the MQV and field point sound pressure curves shift to the right with the increases in the natural frequencies. The MQV curve generally displays the diminishing trend and the total level at the whole range decreases, owing to the enhanced stiffness of the sandwich by the harder core material. The sound pressure curve also declines, especially at the higher frequency. The total level of sound pressure at the whole range 1–500 Hz decreases from 126.26 dB with PVC-H100 to 121.74 dB with PVC-H250, and to 122.52 dB with PVC-H320.

#### 4.1.2. Effect of the Cavity Diameter

For the orthogonal rib-stiffened PVC foam sandwich structures with reinforcing and weakening phases, three typical cavity column diameters, namely 20 mm, 30 mm, and 40 mm, are designed to investigate their effects on the underwater dynamic characteristics. As presented in Table 8, the cavity diameter is negatively correlated with the underwater natural frequencies of the sandwich structure. Increasing the cavity diameter simultaneously reduces both the overall stiffness and mass of the PVC foam core layer. Nevertheless, the variation in natural frequencies is mainly dominated by the reduction in structural stiffness, which outweighs the effect of the mass reduction and thus, leads to a decrease in natural frequencies. Furthermore, the discrepancies in natural frequencies among the three different cavity diameter configurations become increasingly significant with the increase in frequency, exhibiting more pronounced differences in the high-frequency region.

**Figure 9:**
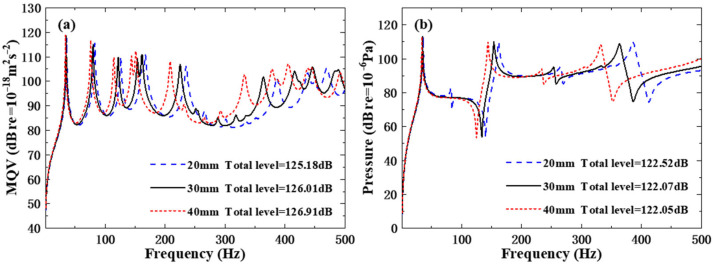
Underwater forced vibration response of sandwich plates with different cavity diameters: (**a**) MQV; (**b**) field point sound pressure.

Figure 9 shows the MQV and field point sound pressure curves of the structure under different cavity diameter schemes. From the above discussion, it can be concluded that variations in the cavity diameter give rise to corresponding changes in the structural natural frequencies, which is then manifested as a curve shift in the acoustic vibration curve graph, especially in the high-frequency range where this shift phenomenon is more obvious. The total level of the MQV rose from 125.18 dB with a cavity diameter of 20 mm to 126.01 dB with a cavity diameter of 30 mm, and to 126.91 dB with a cavity diameter of 40 mm, whereas the total level of field point sound pressure decreased.

#### 4.1.3. Effect of Reinforcing Column Diameter

The diameters of the reinforcing columns are modified successively as 5 mm, 10 mm, and 15 mm. Table 9 lists the influence of the different diameters of reinforcing columns on the underwater natural frequencies of the structure. Analysis shows that under the same mode, the larger the diameter of the reinforcing column, the greater the underwater natural frequencies of the structure. Because increasing the diameter of the reinforcing column will enhance the overall stiffness and mass of the structure within this frequency range, the effect of structural stiffness on natural frequencies predominates over other influencing factors; however, from an overall perspective, fluctuations in the natural frequencies of the rib-stiffened structures under the three different diameter reinforcement column schemes are not particularly significant.

**Figure 10:**
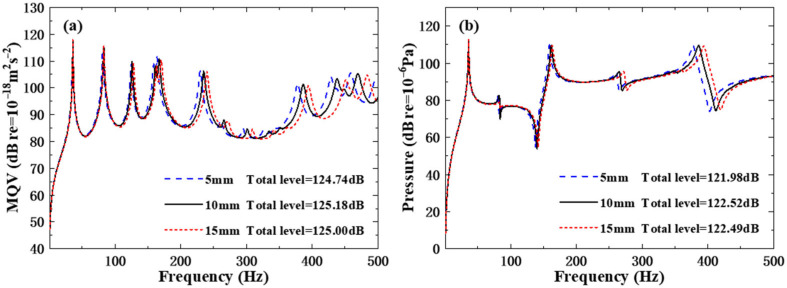
Underwater forced vibration response of sandwich plates with different reinforcing column diameters: (**a**) MQV; (**b**) field point sound pressure.

As can be seen in Figure 10, the MQV curves and the field point sound pressure curves under three different enhanced column diameter schemes almost coincide in the low-frequency region. Only in the high-frequency region can the curve shift be observed, though the amplitude of the shift is not very large. By observing the overall level data of the curve, it can be found that the difference in the total levels among the three schemes does not exceed 0.6 dB, indicating that changing the diameter of the reinforcing column does not have a particularly significant impact on the MQV and the field point sound pressure of the structure 

#### 4.1.4. Effect of Structural Damping

**Figure 11:**
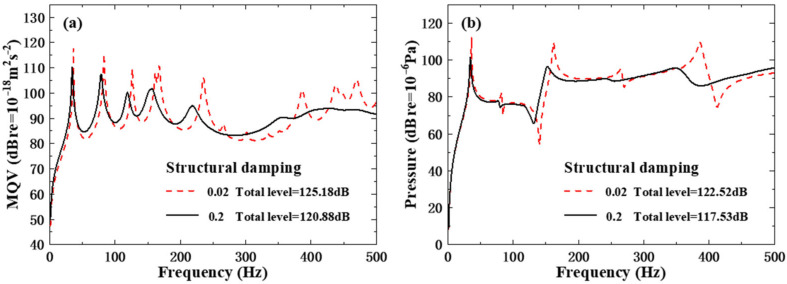
Underwater forced vibration response of sandwich plates with different structural damping: (**a**) MQV; (**b**) field point sound pressure.

The influence of structural damping on the vibro-acoustic characteristics of composite structures is systematically investigated in consideration of two representative damping coefficients, 0.02 and 0.2. As illustrated in Figure 11, structural damping exerts a significant modulation effect on both sthe tructural vibration and underwater acoustic radiation. For the low-damping condition, sharp and prominent resonant peaks are observed in both the MQV and field point sound pressure curves, with the total vibration level reaching 125.18 dB and the total sound pressure level reaching 122.52 dB. In contrast, increasing the structural damping to 0.2 effectively suppresses the resonant amplitudes across the entire frequency range, resulting in a 4.30 dB reduction in the total MQV level and a 4.99 dB reduction in the total field point sound pressure level. This quantitative comparison fully demonstrates that structural damping is a critical parameter for vibration and noise reduction design of underwater composite structures, where appropriately increasing the damping coefficient can achieve remarkable noise reduction performance.

### 4.2. Influence of Rib Parameters

#### 4.2.1. Effect of Rib Material

The ribs were fabricated from CFRP, PU, and steel, with material parameters listed in Table 10. Table 11 shows that rib material properties significantly affect the underwater natural frequencies of the structure. The natural frequencies of the CFRP and steel rib schemes are very close, while those of the PU rib scheme are distinctly lower. This is attributed to the similar Young’s modulus of CFRP (230 GPa) and steel (210 GPa), leading to negligible stiffness differences. Despite the much higher density of steel, its mass contribution to the overall structure is limited. In contrast, the far lower Young’s modulus of PU causes a significant reduction in structural stiffness K and the natural frequency parameter $\sqrt{K/M}$, resulting in substantially lower natural frequencies.

**Figure 12:**
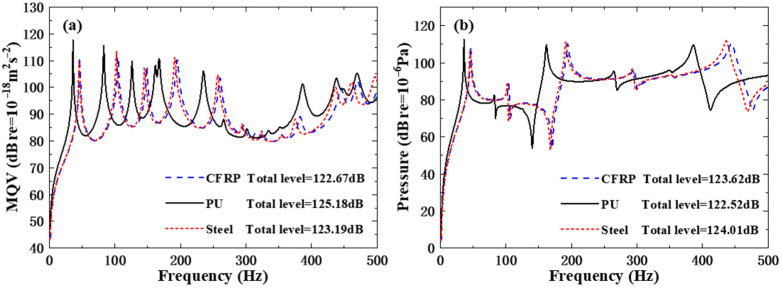
Underwater forced vibration response of sandwich plates with different rib materials: (**a**) MQV; (**b**) field point sound pressure.

By observing Figure 12, it can be seen that for the structure with CFRP and steel ribs, whether it is the MQV curve or the field point sound pressure curve, the peak positions of the curves almost overlap, and their shapes are also highly similar. This is a direct manifestation of the close natural frequencies of the two, indicating that their vibration and acoustic resonance characteristics are basically the same. The peak value of the structural response curve of the rib material PU is concentrated in the lower-frequency region, which is significantly staggered from the peak distribution of CFRP and steel. This is the result of the relatively low natural frequency of the PU, which reflects that the frequency band characteristics of its response are significantly different from the previous two. The total level values of the three are close (CFRP total level = 122.67 dB; PU total level = 125.18 dB; steel total level = 123.19 dB), indicating that the overall response intensity difference is small, though the frequency distribution characteristics of the response are significantly different.

#### 4.2.2. Effect of Rib Widths

The underwater natural frequencies of the orthogonal rib-stiffened PVC foam sandwich structures with reinforcing and weakening phases are calculated by varying the rib width as 10 mm, 20 mm, and 30 mm, respectively, with the results presented in Table 12. The data demonstrate a significant positive correlation between the rib width and the underwater natural frequencies of the structure: as the rib width increases, the natural frequencies of the structure increase monotonically across all analyzed modes. Increasing the rib width simultaneously enhances both the overall structural stiffness and its mass; however, within the analyzed frequency range of 1–500 Hz, the influence of stiffness on the natural frequencies is significantly more pronounced than that of mass, resulting in a net increase in the natural frequencies with the increase in rib width.

**Figure 13:**
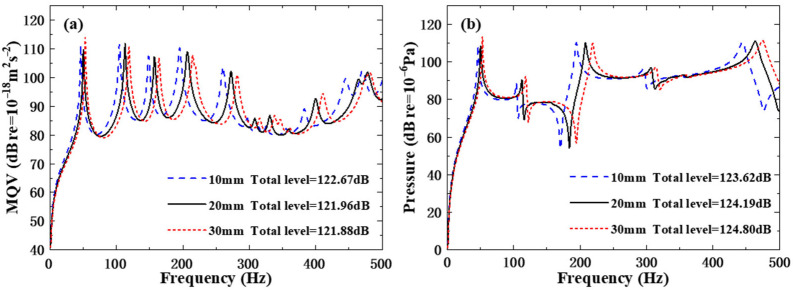
Underwater forced vibration response of sandwich plates with different widths of ribs: (**a**) MQV; (**b**) field point sound pressure.

As the natural frequencies of the structure are in direct proportion to the rib width, the MQV curves and field point sound pressure curves of the three different rib width schemes in Figure 13 mainly show that the characteristic peaks have shifted. The overall level of MQV shows a decreasing trend (10 mm total level = 122.67 dB; 20 mm total level = 121.96 dB; 30 mm total level = 121.88 dB), but the overall level of field point sound pressure shows an increasing trend (10 mm total level = 123.62 dB; 20 mm total level = 124.19 dB; 30 mm total level = 124.80 dB). It indicates that although the increase in rib width suppresses vibration, the overall sound pressure intensity at the field point rises instead, and the conversion efficiency of vibration energy to sound radiation changes with the variation in rib width.

#### 4.2.3. Effect of Rib Height

The height of the ribs was varied as 20 mm, 30 mm, and 40 mm in sequence. The data analysis in Table 13 indicates that the influencing trend of rib height on the underwater natural frequency of the structure was the same as that of width, and both are in direct proportion. Both the global stiffness and total mass of the structure increase simultaneously with an elevation in rib height; however, since the influence of stiffness is dominant within this frequency range, it has manifested as the higher the rib height, the greater the natural frequency under the same mode.

**Figure 14:**
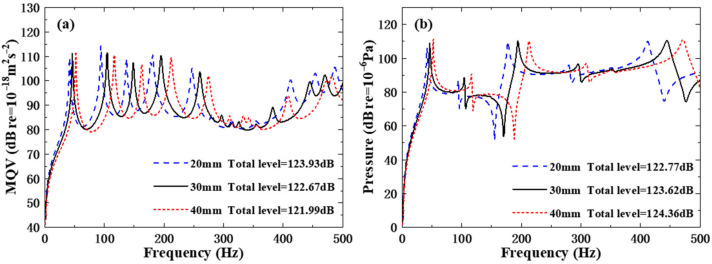
Underwater forced vibration response of sandwich plates with different rib heights: (**a**) MQV; (**b**) field point sound pressure.

Figure 14 shows the MQV curves and field point sound pressure curves of the structures across three different rib height schemes, as well as their total levels. Within the frequency interval of 1–500 Hz, the data indicate that both the MQV curve and the field point sound pressure curve have experienced a shift in their peak values due to the increase in rib height. As the rib height increases, the total level of MQV shows a downward trend, but the total level of sound pressure gradually rises. Because the rib height increases, the stiffness of the structure becomes greater. Under the same external excitation, the vibration displacement and velocity amplitude of the structure will relatively decrease. The increase in the rib height of the field point sound pressure curve causes the resonance peak to shift to the right, slightly increasing the acoustic radiation efficiency of structural vibration and leading to a gradual rise in the total sound pressure level.

### 4.3. Anova-like Contribution Analysis

To quantify the influence weights of each design parameter on the dynamic performance of the structure, based on the principle of single-factor analysis of variance (ANOVA), the sum of squared deviations of each parameter is calculated and its proportion to the total sum of squared deviations is defined as the contribution degree, thereby identifying the key parameters.

For each design parameter j with three levels (j=A,B,…,F), the sum of squared deviations $S_{j}$ is obtained by squaring the differences between each level’s response value and its mean:

(58) $$S_{j}=\sum_{i=1}^{3}{\left(x_{ji}-{\bar{x}}_{j}\right)}^{2}$$

where ${\bar{x}}_{j}$ represents the mean response value of parameter j for the three levels; and the total deviation squared sum $S_{T}$ is the sum of the squared deviations of all parameters. The final contribution degree $\eta_{j}$ is calculated as follows:

(59) $$\eta_{j}=\frac{S_{j}}{S_{T}}\times 100\%$$

Based on the total level data obtained from the previous test in Section 4, the squared sum of deviations and the contribution degree of various parameters of the MQV and the sound pressure at the field points were calculated. The results are shown in the Table 14.

From the Table 14, it can be seen that the core material is the most crucial factor affecting the level of structural vibration with a contribution rate of 47.28%, followed by the material of ribs (22.50%) and then the height of ribs (12.42%). The combined contribution rate of these three parameters exceeds 80%, making them the key design variables for controlling the magnitude of structural vibrations. In addition, the core material also plays a decisive role in the sound radiation performance, with a contribution rate of up to 83.15%. This indicates that the acoustic impedance characteristics of the core material are the dominant factor determining the amount of noise radiated outward by the structure. The secondary influencing factors are the height of ribs (9.01%) and the material of ribs (8.51%), while the contributions of the cavity diameter and the reinforcing diameter column are both less than 2%, having a negligible impact on the overall sound pressure level.

## 5. Conclusions

This paper proposed and deduced a novel semi-analytical method for calculating the underwater vibration response of orthogonal rib-stiffened PVC foam sandwich structures with both reinforcing and weakening phases based on the ESL theory. This method was employed to efficiently predict the underwater natural frequencies, MQV, and field point sound pressure of the target structure. The numerical results were systematically compared with both the published literature data and FE/BE numerical solutions, and excellent agreement was achieved, which rigorously validated the accuracy, reliability, and engineering applicability of the presented method. Furthermore, to reveal the unique underwater acoustic vibration response laws and regulation mechanisms of orthogonal rib-stiffened PVC foam sandwich structures with reinforcing and weakening phases, parametric studies were carried out by adjusting the rib material and dimensions, as well as the core layer parameters. The main conclusions that were drawn are as follows. When the core layer adopts materials such as PVC-250 and PVC-320, the overall levels of structural MQV and field point sound pressure can be remarkably reduced simultaneously. Increasing the cavity diameter leads to an increase in the total MQV level but a decrease in the total field point sound pressure level. In contrast, increasing the diameter of the reinforcing columns reduces the total MQV level, but it results in an elevated total field point sound pressure level. The rib material properties exert a pronounced regulatory effect on the acoustic vibration response characteristics of the structure, and its selection should comprehensively consider key indicators such as stiffness and density. The increase in the rib height and width shows a consistent influence trend, as both can lead to a decrease in the total MQV level and a corresponding increase in the total field point sound pressure level. The fluid–solid coupling model of the orthogonal rib-stiffened PVC foam sandwich structures with both reinforcing and weakening phases established in this paper only takes into account the additional mass effect of water, and it does not calculate acoustic damping and radiation loss. Future work can introduce radiation impedance, structural damping, and fluid damping, with further studies on the underwater vibration response of the structure and the acoustic radiation characteristics.

## Figures and Tables

**Table 1:** Equivalent elastic constants after two step Mori–Tanaka homogenization.

	First Equivalence	Second Equivalence
$E_{11}$ (GPa)	0.461	0.475
$E_{22}$ (GPa)	0.461	0.475
$E_{33}$ (GPa)	0.527	1.72
$G_{12}$ (GPa)	0.137	0.141
$G_{13}$ (GPa)	0.145	0.152
$G_{23}$ (GPa)	0.145	0.152
Mass density (kg/m^3^)	305.92	313.46
Structural damping	0.02	0.02

**Table 2:** First few natural frequencies of (*m*, *n*) modes for the composite structure in water.

(*m*, *n*)	Natural Frequencies (Hz)					
	n	n = 1	n = 2	n = 3	n = 4	n = 5
In water	m = 1	35.75	126.10	267.05	440.20	635.52
	m = 2	82.78	167.59	302.16	471.83	662.00
	m = 3	161.76	235.59	352.26	511.43	690.18
	m = 4	266.13	335.44	443.22	608.37	790.69
	m = 5	388.11	450.91	541.62	703.72	875.16
	m = 6	523.92	578.55	650.05	806.59	958.02

**Table 3:** ANSYS meshes convergence verification for different (*m*, *n*) modes.

Mode Half-Wave Numbers		Number of ANSYS Meshes		
*m*	*n*	238,152	365,640	Difference (%)
1	1	35.75	35.90	0.42
1	2	126.10	126.78	0.54
1	3	267.05	268.12	0.40
2	1	82.78	82.70	0.10
2	2	167.59	167.80	0.13
2	3	302.16	302.74	0.20
3	1	161.76	161.60	0.10
3	2	235.59	235.48	0.05
3	3	352.26	352.45	0.05

**Table 4:** Acoustic mesh convergence verification for different (*m*, *n*) modes.

Mode Half-Wave Numbers		Number of Acoustic Meshes		
*m*	*n*	7420	11,280	Difference (%)
1	1	35.75	35.74	0.03
1	2	126.10	125.90	0.16
1	3	267.05	266.10	0.36
2	1	82.78	82.74	0.05
2	2	167.59	167.28	0.18
2	3	302.16	301.02	0.38
3	1	161.76	161.48	0.17
3	2	235.59	234.94	0.28
3	3	352.26	350.70	0.44

**Table 5:** First five natural frequencies: theoretical predictions versus numerical results for simply supported structures in vacuum and water.

Cases of Ribs	(***m***, ***n***)	Natural Frequencies (Hz)						
		In Vacuum ($f_{v}$)			In Water ($f_{w}$)			$f_{v}/f_{w}$
		Analytical	ANSYS	Errors	Analytical	Virtual.Lab	Errors	
*n*_1_ = 1 *n*_2_ = 2	(1, 1)	151.47	154.56	2.04%	35.66	35.52	0.39%	4.25
	(2, 1)	281.68	287.82	2.13%	80.72	81.56	1.04%	3.49
	(1, 2)	417.38	409.94	1.81%	129.20	125.81	2.62%	3.23
	(3, 1)	478.30	484.23	1.22%	157.34	158.80	0.93%	3.04
	(2, 2)	485.22	496.93	2.36%	162.23	165.36	1.93%	2.99
*n*_1_ = 2 *n*_2_ = 2	(1, 1)	151.89	154.73	2.03%	35.87	35.75	0.33%	4.23
	(2, 1)	288.40	290.70	0.80%	82.92	82.78	0.14%	3.48
	(1, 2)	414.71	405.89	2.13%	129.89	126.10	2.93%	3.19
	(3, 1)	496.93	490.79	1.24%	164.08	161.76	1.41%	3.03
	(2, 2)	498.47	497.83	0.13%	168.91	167.59	0.78%	2.95
*n*_1_ = 2 *n*_2_ = 3	(1, 1)	153.92	155.60	1.09%	35.92	36.02	0.25%	4.29
	(2, 1)	289.21	290.72	0.52%	83.01	82.94	0.08%	3.48
	(1, 2)	426.33	409.83	3.87%	131.52	127.77	2.85%	3.24
	(3, 1)	497.18	486.09	2.23%	164.47	162.06	1.47%	3.02
	(2, 2)	508.55	501.07	1.47%	170.65	169.05	0.94%	2.98

**Table 6:** Material parameters of the core layer.

Core Layer Material	PVC-H100	PVC-H250	PVC-H320
Young’s modulus (MPa)	135	300	450
Poisson’s ratio	0.3	0.3	0.3
Mass density (kg/m^3^)	100	250	320
Structural damping	0.02	0.02	0.02

**Table 7:** Underwater natural frequencies of rib-stiffened PVC foam sandwich structures with reinforcing and weakening phases using different core layer materials (Hz).

Mode Half-Wave Numbers		Core Layer Materials		
*m*	*n*	PVC-H100	PVC-H250	PVC-H320
1	1	31.10	34.37	35.75
1	2	97.08	116.60	126.10
1	3	190.55	239.67	267.05
2	1	67.20	77.91	82.78
2	2	126.31	153.71	167.59
2	3	213.64	270.25	302.16
3	1	122.63	148.55	161.76
3	2	171.44	213.21	235.59
3	3	243.18	312.54	352.26

**Table 8:** Underwater natural frequencies of rib-stiffened PVC foam sandwich structures with reinforcing and weakening phases using different cavity diameters (Hz).

Mode Half-Wave Numbers		Cavity Diameters (Volume Fraction)		
*m*	*n*	20 mm (6.21%)	30 mm (13.96%)	40 mm (24.82%)
1	1	35.75	34.83	33.86
1	2	126.10	121.86	114.62
1	3	267.05	255.45	235.14
2	1	82.78	79.67	76.08
2	2	167.59	161.68	151.43
2	3	302.16	288.99	265.67
3	1	161.76	154.12	144.17
3	2	235.59	225.67	209.16
3	3	352.26	335.98	306.89

**Table 9:** Underwater natural frequencies of rib-stiffened PVC foam sandwich structures with reinforcing and weakening phases using different reinforcing column diameter (Hz).

Mode Half-Wave Numbers		Reinforcing Diameters (Volume Fraction)		
*m*	*n*	5 mm (0.44%)	10 mm (1.75%)	15 mm (3.93%)
1	1	35.29	35.75	35.79
1	2	124.66	126.10	128.39
1	3	262.67	267.05	274.13
2	1	81.45	82.78	83.08
2	2	164.38	167.59	170.71
2	3	295.83	302.16	310.35
3	1	158.99	161.76	163.54
3	2	230.23	235.59	240.50
3	3	343.50	352.26	362.55

**Table 10:** Material parameters of the ribs.

Materials of Ribs	CFRP	PU	Steel
Young’s modulus (GPa)	230	14	210
Shear modulus(GPa)	/	/	80
Poisson’s ratio	0.25	0.28	0.3
Mass density (kg/m^3^)	1800	760	7850

**Table 11:** Underwater natural frequencies of rib-stiffened PVC foam sandwich structures with reinforced and weakened phases using different rib materials (Hz).

Mode Half-Wave Numbers		Materials of Ribs		
*m*	*n*	CFRP	PU	Steel
1	1	46.11	35.75	45.20
1	2	148.67	126.10	145.72
1	3	283.19	267.05	295.53
2	1	104.52	82.78	102.27
2	2	195.82	167.59	191.88
2	3	326.86	302.16	324.71
3	1	194.63	161.76	191.70
3	2	261.10	235.59	257.73
3	3	356.06	352.26	355.33

**Table 12:** Underwater natural frequencies of rib-stiffened PVC foam sandwich structures with reinforced and weakened phases using different rib widths (Hz).

Mode Half-Wave Numbers		Widths of Ribs		
*m*	*n*	10 mm	20 mm	30 mm
1	1	46.11	50.17	52.86
1	2	148.67	157.51	164.27
1	3	298.06	309.57	317.04
2	1	104.52	112.77	118.69
2	2	195.82	206.83	215.25
2	3	326.86	337.25	339.31
3	1	194.63	208.65	219.42
3	2	261.10	273.20	282.64
3	3	356.06	359.92	362.23

**Table 13:** Underwater natural frequencies of rib-stiffened PVC foam sandwich structures with reinforced and weakened phases using different rib heights (Hz).

Mode Half-Wave Numbers		Heights of Ribs		
*m*	*n*	20 mm	30 mm	40 mm
1	1	41.38	46.11	51.54
1	2	137.06	148.67	162.91
1	3	281.74	298.06	311.85
2	1	94.17	104.52	116.56
2	2	181.48	195.82	212.45
2	3	314.52	326.86	334.61
3	1	177.67	194.63	213.25
3	2	247.66	261.10	275.54
3	3	354.88	356.06	356.94

**Table 14:** The sum of squared deviations of each parameter and the analysis results of contribution degree.

		MQV		Pressure	
Number	Design parameter	$S_{j}$	$\eta_{j}$ (%)	$S_{j}$	$\eta_{j}$ (%)
A	Core material	7.373	47.28	11.662	83.15
B	Cavity diameter	1.498	9.61	0.142	1.01
C	Reinforcing diameter	0.098	0.63	0.183	1.31
D	Material of ribs	3.509	22.50	1.193	8.51
E	Width of ribs	0.378	2.42	0.696	4.96
F	Height of ribs	1.937	12.42	1.263	9.01
	$S_{T}$	15.595	100.00	14.039	100.00

## Data Availability

The raw/processed data required to reproduce these findings cannot be shared at this time due to legal or ethical reasons.

## Appendix A

Motion equations of the submerged rib-stiffened sandwich are as follows:

(A1) $$\begin{array}{ll} \delta u_{0}: & \left(A_{11}u_{0,x,x}+A_{66}u_{0,y,y}\right)+\left(A_{12}+A_{66}\right)v_{0,x,y}+\left(B_{11}\theta_{x,x,x}+B_{66}\theta_{x,y,y}\right)\;+\left(B_{12}+B_{66}\right)\theta_{y,x,y}\\ & +\sum_{j=1}^{n_{1}}b_{j}A_{11}^{bj}\left(u_{0,x,x}\;-e_{j}\theta_{x,x,x}\right)\bar{\delta }(y-y_{j})\;-\left({\bar{\rho }}_{0}{\ddot{u}}_{0}+{\bar{\rho }}_{1}{\ddot{\theta }}_{x}\right)-\sum_{j=1}^{n_{1}}b_{j}\left({\bar{\rho }}_{x0}^{bj}{\ddot{u}}_{0}+{\bar{\rho }}_{x1}^{bj}{\ddot{\theta }}_{x}\right)\bar{\delta }(y-y_{j})\;=0 \end{array}$$

(A2) $$\begin{array}{ll} \delta v_{0}: & \left(A_{12}+A_{66}\right)u_{0,x,y}+\left(A_{66}v_{0,x,x}+A_{22}v_{0,y,y}\right)+\left(B_{12}+B_{66}\right)\theta_{x,x,y}+\left(B_{66}\theta_{y,x,x}+B_{22}\theta_{y,y,y}\right)\\ & +\sum_{i=1}^{n_{2}}b_{i}A_{22}^{bi}\left(v_{0,y,y}-e_{i}\theta_{y,y,y}\right)\bar{\delta }(x-x_{i})\;-\left({\bar{\rho }}_{0}{\ddot{v}}_{0}+{\bar{\rho }}_{1}{\ddot{\theta }}_{y}\right)-\sum_{i=1}^{n_{2}}b_{i}\left({\bar{\rho }}_{y0}^{bi}{\ddot{v}}_{0}+{\bar{\rho }}_{y1}^{bi}{\ddot{\theta }}_{y}\right)\bar{\delta }(x-x_{i})=0 \end{array}$$

(A3) $$\begin{array}{ll} \delta \theta_{x}: & \left(B_{11}u_{0,x,x}+B_{66}u_{0,y,y}\right)+\left(B_{12}+B_{66}\right)v_{0,x,y}\;\;+\left(D_{11}\theta_{x,x,x}+D_{66}\theta_{x,y,y}\right)-C_{55}\theta_{x}-\sum_{j=1}^{n_{1}}b_{j}C_{55}^{bj}\theta_{x}\bar{\delta }(y-y_{j})\;\;\;\;\;\\ & +\left(D_{12}+D_{66}\right)\theta_{y,x,y}\;\;-C_{55}w_{0,x}+\sum_{j=1}^{n_{1}}b_{j}\left[D_{11}^{bj}\theta_{x,x,x}-e_{j}A_{11}^{bj}\left(u_{0,x,x}-e_{j}\theta_{x,x,x}\right)-C_{55}^{bj}w_{0,x}\right]\bar{\delta }(y-y_{j})\;\\ & -\left({\bar{\rho }}_{1}{\ddot{u}}_{0}+{\bar{\rho }}_{2}{\ddot{\theta }}_{x}\right)-\sum_{j=1}^{n_{1}}b_{j}\left({\bar{\rho }}_{x1}^{bj}{\ddot{u}}_{0}+{\bar{\rho }}_{x2}^{bj}{\ddot{\theta }}_{x}\right)\bar{\delta }(y-y_{j})\;=0 \end{array}$$

(A4) $$\begin{array}{ll} \delta \theta_{y}: & \left(B_{12}+B_{66}\right)u_{0,x,y}+\left(B_{66}v_{0,x,x}+B_{22}v_{0,y,y}\right)\;+\left(D_{66}\theta_{y,x,x}+D_{22}\theta_{y,y,y}\right)-C_{44}\theta_{y}-\sum_{i=1}^{n_{2}}b_{i}C_{44}^{bi}\theta_{y}\bar{\delta }(x-x_{i})\\ & +\left(D_{12}+D_{66}\right)\theta_{x,x,y}-C_{44}w_{0,y}+\sum_{i=1}^{n_{2}}b_{i}\left[D_{22}^{bi}\theta_{y,y,y}-e_{i}A_{22}^{bi}\left(v_{0,y,y}-e_{i}\theta_{y,y,y}\right)-C_{44}^{bi}w_{0,y}\right]\bar{\delta }(x-x_{i})\\ & -\left({\bar{\rho }}_{1}{\ddot{v}}_{0}+{\bar{\rho }}_{2}{\ddot{\theta }}_{y}\right)-\sum_{i=1}^{n_{2}}b_{i}\left({\bar{\rho }}_{y1}^{bi}{\ddot{v}}_{0}+{\bar{\rho }}_{y2}^{bi}{\ddot{\theta }}_{y}\right)\bar{\delta }(x-x_{i})=0 \end{array}$$

(A5) $$\delta w_{0}:-\int_{\Omega }\left[\begin{array}{ll} C_{55}\theta_{x,x} & +C_{55}w_{0,x,x}-{\bar{\rho }}_{0}{\ddot{w}}_{0}+C_{44}\theta_{y,y}+C_{44}w_{0,y,y}+q_{z}\\ & +\sum_{j=1}^{n_{1}}b_{j}\left(C_{55}^{bj}\theta_{x,x}+C_{55}^{bj}w_{0,x,x}-{\bar{\rho }}_{x0}^{bj}{\ddot{w}}_{0}\right)\bar{\delta }(y-y_{j})\;\\ & +\sum_{i=1}^{n_{2}}b_{i}\left(C_{44}^{bi}\theta_{y,y}+C_{44}^{bi}w_{0,y,y}-{\bar{\rho }}_{y0}^{bi}{\ddot{w}}_{0}\right)\bar{\delta }(x-x_{i})\; \end{array}\right]{\left.\cdot \frac{\partial^{2}G}{\partial z_{M}\partial z_{0}}\right|}_{z_{M},z_{0}=0}dxdy=\rho_{0}\omega^{2}w_{0}$$

## Appendix B

The coefficients in Equation (48) are as follows:

(A6) $$\left\{\begin{array}{l} {\bar{R}}_{11}=-\left(A_{11}\alpha^{2}+A_{66}\beta^{2}-{\bar{\rho }}_{0}\omega^{2}\right)-\sum_{j=1}^{n_{1}}b_{j}\left(A_{11}^{bj}\alpha^{2}-{\bar{\rho }}_{x0}^{bj}\omega^{2}\right)\cdot \kappa (j)\;\;\;{\bar{R}}_{12}=-\alpha \beta \left(A_{12}+A_{66}\right)\\ {\bar{R}}_{13}=-\left(B_{11}\alpha^{2}+B_{66}\beta^{2}-{\bar{\rho }}_{1}\omega^{2}\right)+\sum_{j=1}^{n_{1}}b_{j}\left(e_{j}A_{11}^{bj}\alpha^{2}+{\bar{\rho }}_{x1}^{bj}\omega^{2}\right)\cdot \kappa (j)\;\;\;{\bar{R}}_{14}=-\alpha \beta \left(B_{12}+B_{66}\right)\;\;\;{\bar{R}}_{15}=0 \end{array}\right.$$

(A7) $$\left\{\begin{array}{l} {\bar{R}}_{21}=-\alpha \beta \left(A_{12}+A_{66}\right)\;\;\;{\bar{R}}_{22}=-\left(A_{66}\alpha^{2}+A_{22}\beta^{2}-{\bar{\rho }}_{0}\omega^{2}\right)-\sum_{i=1}^{n_{2}}b_{i}\left(A_{22}^{bi}\beta^{2}-{\bar{\rho }}_{y0}^{bi}\omega^{2}\right)\cdot \iota (i)\\ {\bar{R}}_{23}=-\alpha \beta \left(B_{12}+B_{66}\right)\;\;\;{\bar{R}}_{24}=-\left(B_{66}\alpha^{2}+B_{22}\beta^{2}-{\bar{\rho }}_{1}\omega^{2}\right)+\sum_{i=1}^{n_{2}}b_{i}\left(e_{i}A_{22}^{bi}\beta^{2}+{\bar{\rho }}_{y1}^{bi}\omega^{2}\right)\cdot \iota (i)\;\;\;{\bar{R}}_{25}=0 \end{array}\right.$$

(A8) $$\left\{\begin{array}{l} {\bar{R}}_{31}=-\left(B_{11}\alpha^{2}+B_{66}\beta^{2}-{\bar{\rho }}_{1}\omega^{2}\right)+\sum_{j=1}^{n_{1}}b_{j}\left(e_{j}A_{11}^{bj}\alpha^{2}+{\bar{\rho }}_{x1}^{bj}\omega^{2}\right)\cdot \kappa (j)\;\;\;{\bar{R}}_{32}=-\alpha \beta \left(B_{12}+B_{66}\right)\\ {\bar{R}}_{33}=-\left(D_{11}\alpha^{2}+D_{66}\beta^{2}+C_{55}-{\bar{\rho }}_{2}\omega^{2}\right)\;-\sum_{j=1}^{n_{1}}b_{j}\left(D_{11}^{bj}\alpha^{2}+e_{j}^{2}A_{11}^{bj}\alpha^{2}+C_{55}^{bj}-{\bar{\rho }}_{x2}^{bj}\omega^{2}\right)\cdot \kappa (j)\\ {\bar{R}}_{34}=-\alpha \beta \left(D_{12}+D_{66}\right)\;\;\;{\bar{R}}_{35}=-\alpha C_{55}-\alpha \sum_{j=1}^{n_{1}}b_{j}C_{55}^{bj}\cdot \kappa (j) \end{array}\right.$$

(A9) $$\left\{\begin{array}{l} {\bar{R}}_{41}=-\alpha \beta \left(B_{12}+B_{66}\right)\;\;\;{\bar{R}}_{42}=-\left(B_{66}\alpha^{2}+B_{22}\beta^{2}-{\bar{\rho }}_{1}\omega^{2}\right)\;+\sum_{i=1}^{n_{2}}b_{i}\left(e_{i}A_{22}^{bi}\beta^{2}+{\bar{\rho }}_{x1}^{bi}\omega^{2}\right)\cdot \iota (i)\\ {\bar{R}}_{43}=-\alpha \beta \left(D_{12}+D_{66}\right)\;\;\;{\bar{R}}_{45}=-\beta C_{44}-\beta \sum_{i=1}^{n_{2}}b_{i}C_{44}^{bi}\cdot \iota (i)\\ {\bar{R}}_{44}=-\left(D_{66}\alpha^{2}+D_{22}\beta^{2}+C_{44}-{\bar{\rho }}_{2}\omega^{2}\right)-\sum_{i=1}^{n_{2}}b_{i}\left(D_{22}^{bi}\beta^{2}+e_{i}^{2}A_{22}^{bi}\beta^{2}+C_{44}^{bi}-{\bar{\rho }}_{y2}^{bi}\omega^{2}\right)\cdot \iota (i) \end{array}\right.$$

(A10) $$\left\{\begin{array}{l} {\bar{R}}_{51}=0\;\;\;{\bar{R}}_{52}=0\\ {\bar{R}}_{53}=-\alpha C_{55}\cdot \gamma_{mn\;mn}-\alpha \sum_{j=1}^{n_{1}}b_{j}C_{55}^{bj}\cdot \gamma_{mn\;\;mn}^{bj}\;\;\;{\bar{R}}_{54}=-\beta C_{44}\cdot \gamma_{mn\;mn}-\beta \sum_{i=1}^{n_{2}}b_{i}C_{44}^{bi}\cdot \gamma_{mn\;\;mn}^{bi}\\ {\bar{R}}_{55}=-\left(C_{55}\alpha^{2}+C_{44}\beta^{2}-{\bar{\rho }}_{0}\omega^{2}\right)\;\;\cdot \gamma_{mn\;mn}+\rho_{0}\omega^{2}\cdot g(m,m,L_{x})g(n,n,L_{y})\\ \;\;\;\;\;\;\;\;\;\;\;-\sum_{j=1}^{n_{1}}b_{j}\left(C_{55}^{bj}\alpha^{2}-{\bar{\rho }}_{x0}^{bj}\omega^{2}\right)\cdot \gamma_{mn\;\;mn}^{bj}-\sum_{i=1}^{n_{2}}b_{i}\left(C_{44}^{bi}\beta^{2}-{\bar{\rho }}_{y0}^{bi}\omega^{2}\right)\cdot \gamma_{mn\;\;mn}^{bi} \end{array}\right.$$

where

(A11) $$\kappa (j)=\frac{2}{L_{y}}\sin^{2}[y_{j}n\pi /L_{y}],\;\;\;\iota (i)=\frac{2}{L_{x}}\sin^{2}[x_{i}m\pi /L_{x}]$$

(A12) $$\begin{array}{l} f(m_{1},m_{2},l_{0})=\int_{0}^{l_{0}}\cos\frac{m_{1}\pi \xi }{l_{0}}\cos\frac{m_{2}\pi \xi }{l_{0}}d\xi =\left\{\begin{array}{c} 0\;\;,\;\;m_{1}\neq m_{2}\\ l_{0}/2\;,m_{1}=m_{2} \end{array}\right.\\ g(m_{1},m_{2},l_{0})=\int_{0}^{l_{0}}\sin\frac{m_{1}\pi \xi }{l_{0}}\sin\frac{m_{2}\pi \xi }{l_{0}}d\xi =\left\{\begin{array}{c} 0\;\;,\;\;m_{1}\neq m_{2}\\ l_{0}/2\;,m_{1}=m_{2} \end{array}\right. \end{array}$$

(A13) $$\begin{array}{l} \gamma_{pq\;mn}=\int_{S_{p}}\int_{S_{p}}\sin\frac{m\pi x}{L_{x}}\sin\frac{n\pi y}{L_{y}}\sin\frac{p\pi x}{L_{x}}\sin\frac{q\pi y}{L_{y}}{\left.\frac{\partial^{2}G(M,M_{0})}{\partial z_{M}\partial z_{0}}\right|}_{z_{M},z_{0}=0}dxdydx_{0}dy_{0}\\ =2j_{1}L_{x}^{2}L_{y}^{2}\pi^{2}\int_{0}^{\infty }\int_{0}^{\infty }\frac{pqmn\sqrt{k_{0}^{2}-k_{x}^{2}-k_{y}^{2}}(1-{\left(-1\right)}^{p}\cos k_{x}L_{x})(1-{\left(-1\right)}^{q}\cos k_{y}L_{y})}{\left(L_{x}^{2}k_{x}^{2}-p^{2}\pi^{2})(L_{y}^{2}k_{y}^{2}-q^{2}\pi^{2})(L_{x}^{2}k_{x}^{2}-m^{2}\pi^{2})(L_{y}^{2}k_{y}^{2}-n^{2}\pi^{2}\right)}dk_{x}dk_{y} \end{array}$$

As the parity of *p* and *m* (or *q* and *n*) is different, there is $\gamma_{pq\;mn}^{bj}\;=0$ (or $\gamma_{pq\;\;mn}^{bi}\;=0$). On the contrary, when the parity is same, these are as follows:

(A14) $$\begin{array}{l} \gamma_{pq\;\;mn}^{bj}\;=\int_{S_{p}}\int_{S_{p}}\sin\frac{m\pi x}{L_{x}}\sin\frac{n\pi y}{L_{y}}\bar{\delta }(y-y_{j})\sin\frac{p\pi x_{0}}{L_{x}}\sin\frac{q\pi y_{0}}{L_{y}}\cdot {\left.\frac{\partial^{2}G(M,M_{0})}{\partial z_{M}\partial z_{M_{0}}}\right|}_{z_{M},z_{0}=0}dxdydx_{0}dy_{0}\\ \;=\frac{j_{1}L_{x}^{2}\pi }{2}\int_{-\infty }^{+\infty }\int_{0}^{+\infty }\frac{pqmL_{y}\sqrt{k_{0}^{2}-k_{x}^{2}-k_{y}^{2}}[1-{\left(-1\right)}^{p}\cos k_{x}L_{x}][{\left(-1\right)}^{q}e^{-jk_{y}L_{y}}-1]}{\left(L_{x}^{2}k_{x}^{2}-m^{2}\pi^{2})(L_{x}^{2}k_{x}^{2}-p^{2}\pi^{2})(L_{y}^{2}k_{y}^{2}-q^{2}\pi^{2}\right)}\sin\frac{n\pi y_{j}}{b}e^{jk_{y}y_{j}}dk_{x}dk_{y} \end{array}$$

(A15) $$\begin{array}{l} \gamma_{pq\;\;\;mn}^{bi}=\int_{S_{p}}\int_{S_{p}}\sin\frac{m\pi x}{L_{x}}\sin\frac{n\pi y}{L_{y}}\bar{\delta }(x-x_{i})\sin\frac{k\pi x_{0}}{L_{x}}\sin\frac{l\pi y_{0}}{L_{y}}\cdot {\left.\frac{\partial^{2}G(M,M_{0})}{\partial z_{M}\partial z_{M_{0}}}\right|}_{z_{M},z_{0}=0}dxdydx_{0}dy_{0}\\ \;\;=\frac{j_{1}L_{y}^{2}\pi }{2}\int_{-\infty }^{+\infty }\int_{0}^{+\infty }\frac{pqnL_{x}\sqrt{k_{0}^{2}-k_{x}^{2}-k_{y}^{2}}[1-{\left(-1\right)}^{q}\cos k_{y}L_{y}][{\left(-1\right)}^{p}e^{-jk_{x}L_{x}}-1]}{\left(L_{y}^{2}k_{y}^{2}-q^{2}\pi^{2})(L_{y}^{2}k_{y}^{2}-n^{2}\pi^{2})(L_{x}^{2}k_{x}^{2}-p^{2}\pi^{2}\right)}\;\;\sin\frac{m\pi x_{i}}{L_{x}}e^{jk_{x}x_{i}}dk_{y}dk_{x} \end{array}$$

## Appendix C

The expressions of ${\bar{T}}_{ij}$ and ${\bar{O}}_{pq}$ in Equations (49)–(51) are as follows:

$$\bar{\kappa }(j)=f(p,m,L_{x})\sin^{2}[y_{j}n\pi /L_{y}],\;\;\;\bar{\iota }(i)=\sin^{2}[x_{i}m\pi /L_{x}]f(q,n,L_{y})$$

(A16) $$\left\{\begin{array}{l} {\bar{T}}_{11}=-\left(A_{11}\alpha^{2}+A_{66}\beta^{2}-{\bar{\rho }}_{0}\omega^{2}\right)\cdot \xi_{1}-\sum_{j=1}^{n_{1}}b_{j}\left(A_{11}^{bj}\alpha^{2}-{\bar{\rho }}_{x0}^{bj}\omega^{2}\right)\cdot \bar{\kappa }(j)\\ {\bar{T}}_{13}=-\left(B_{11}\alpha^{2}+B_{66}\beta^{2}-{\bar{\rho }}_{1}\omega^{2}\right)\cdot \xi_{1}+\sum_{j=1}^{n_{1}}b_{j}\left(e_{j}A_{11}^{bj}\alpha^{2}+{\bar{\rho }}_{x1}^{bj}\omega^{2}\right)\cdot \bar{\kappa }(j)\;\;\;{\bar{T}}_{1k}={\bar{R}}_{1k}\cdot \xi_{1}\;\;(k=2,4,5) \end{array}\right.$$

(A17) $$\left\{\begin{array}{l} {\bar{T}}_{22}=-\left(A_{66}\alpha^{2}+A_{22}\beta^{2}-{\bar{\rho }}_{0}\omega^{2}\right)\cdot \xi_{2}-\sum_{i=1}^{n_{2}}b_{i}\left(A_{22}^{bi}\beta^{2}-{\bar{\rho }}_{y0}^{bi}\omega^{2}\right)\cdot \bar{\iota }(i)\\ {\bar{T}}_{24}=-\left(B_{66}\alpha^{2}+B_{22}\beta^{2}-{\bar{\rho }}_{1}\omega^{2}\right)\cdot \xi_{2}+\sum_{i=1}^{n_{2}}b_{i}\left(e_{i}A_{22}^{bi}\beta^{2}+{\bar{\rho }}_{y1}^{bi}\omega^{2}\right)\cdot \bar{\iota }(i)\;\;\;{\bar{T}}_{2k}={\bar{R}}_{2k}\cdot \xi_{2}\;\;(k=1,3,5) \end{array}\right.$$

(A18) $$\left\{\begin{array}{l} {\bar{T}}_{31}=-\left(B_{11}\alpha^{2}+B_{66}\beta^{2}-{\bar{\rho }}_{1}\omega^{2}\right)\cdot \xi_{1}+\sum_{j=1}^{n_{1}}b_{j}\left(e_{j}A_{11}^{bj}\alpha^{2}+{\bar{\rho }}_{x1}^{bj}\omega^{2}\right)\cdot \bar{\kappa }(j)\;\;\;{\bar{T}}_{32}=-\alpha \beta \left(B_{12}+B_{66}\right)\cdot \xi_{1}\\ {\bar{T}}_{33}=-\left(D_{11}\alpha^{2}+D_{66}\beta^{2}+C_{55}-{\bar{\rho }}_{2}\omega^{2}\right)\;\cdot \xi_{1}-\sum_{j=1}^{n_{1}}b_{j}\left(D_{11}^{bj}\alpha^{2}+e_{j}^{2}A_{11}^{bj}\alpha^{2}+C_{55}^{bj}-{\bar{\rho }}_{x2}^{bj}\omega^{2}\right)\cdot \bar{\kappa }(j)\\ {\bar{T}}_{34}=-\alpha \beta \left(D_{12}+D_{66}\right)\cdot \xi_{1}\;\;\;{\bar{T}}_{35}=-\alpha C_{55}\cdot \xi_{1}-\alpha \sum_{j=1}^{n_{1}}b_{j}C_{55}^{bj}\cdot \bar{\kappa }(j) \end{array}\right.$$

(A19) $$\left\{\begin{array}{l} {\bar{T}}_{41}=-\alpha \beta \left(B_{12}+B_{66}\right)\cdot \xi_{2}\;\;\;{\bar{T}}_{42}=-\left(B_{66}\alpha^{2}+B_{22}\beta^{2}-{\bar{\rho }}_{1}\omega^{2}\right)\cdot \xi_{2}+\sum_{i=1}^{n_{2}}b_{i}\left(e_{i}A_{22}^{bi}\beta^{2}+{\bar{\rho }}_{x1}^{bi}\omega^{2}\right)\cdot \bar{\iota }(i)\\ {\bar{T}}_{43}=-\alpha \beta \left(D_{12}+D_{66}\right)\cdot \xi_{2}\;\;\;{\bar{T}}_{45}=-\beta C_{44}\cdot \xi_{2}-\beta \sum_{i=1}^{n_{2}}b_{i}C_{44}^{bi}\cdot \bar{\iota }(i)\\ {\bar{T}}_{44}=-\left(D_{66}\alpha^{2}+D_{22}\beta^{2}+C_{44}-{\bar{\rho }}_{2}\omega^{2}\right)\cdot \xi_{2}-\sum_{i=1}^{n_{2}}b_{i}\left(D_{22}^{bi}\beta^{2}+e_{i}^{2}A_{22}^{bi}\beta^{2}+C_{44}^{bi}-{\bar{\rho }}_{y2}^{bi}\omega^{2}\right)\cdot \bar{\iota }(i) \end{array}\right.$$

(A20) $$\left\{\begin{array}{l} {\bar{T}}_{51}=0\;\;\;{\bar{T}}_{52}=0\\ {\bar{T}}_{53}=-\alpha C_{55}\cdot \gamma_{pq\;mn}\;-\alpha \sum_{j=1}^{n_{1}}b_{j}C_{55}^{bj}\cdot \gamma_{pq\;mn}^{bj}\;\;\;{\bar{T}}_{54}=-\beta C_{44}\cdot \gamma_{pq\;mn}\;-\beta \sum_{i=1}^{n_{2}}b_{i}C_{44}^{bi}\cdot \gamma_{pq\;mn}^{bi}\\ {\bar{T}}_{55}=-\left(C_{55}\alpha^{2}+C_{44}\beta^{2}-{\bar{\rho }}_{0}\omega^{2}\right)\;\cdot \gamma_{pq\;mn}+\rho_{0}\omega^{2}\cdot \xi_{3}\\ \;\;\;\;\;\;\;\;\;\;\;-\sum_{j=1}^{n_{1}}b_{j}\left(C_{55}^{bj}\alpha^{2}-{\bar{\rho }}_{x0}^{bj}\omega^{2}\right)\cdot \gamma_{pq\;mn}^{bj}-\sum_{i=1}^{n_{2}}b_{i}\left(C_{44}^{bi}\beta^{2}-{\bar{\rho }}_{y0}^{bi}\omega^{2}\right)\cdot \gamma_{pq\;mn}^{bi} \end{array}\right.$$

where

(A21) $$\bar{\kappa }(j)=f(p,m,L_{x})\sin^{2}[y_{j}n\pi /L_{y}],\;\;\;\bar{\iota }(i)=\sin^{2}[x_{i}m\pi /L_{x}]f(q,n,L_{y})$$

(A22) $$\left\{\begin{array}{l} \xi_{1}=f(p,m,L_{x})g(q,n,L_{y})\\ \xi_{2}=g(p,m,L_{x})f(q,n,L_{y})\\ \xi_{3}=g(p,m,L_{x})g(q,n,L_{y}) \end{array}\right.$$
